# XAF1 directs glioma response to temozolomide through apoptotic transition of autophagy by activation of ATM–AMPK signaling

**DOI:** 10.1093/noajnl/vdac013

**Published:** 2022-02-07

**Authors:** Min-Goo Lee, Zisun Choi, Na-Jung Lim, Ji-Sun Lim, Kyung-Woo Lee, Kyung-Phil Ko, Byung-Kyu Ryu, Shin-Hyuk Kang, Sung-Gil Chi

**Affiliations:** 1 Department of Life Sciences, Korea University, Seoul, Republic of Korea; 2 Department of Neurosurgery, School of Medicine, Korea University, Seoul, Republic of Korea

**Keywords:** AMPK, apoptosis, glioblastoma, temozolomide, XAF1

## Abstract

**Background:**

X-linked inhibitor of apoptosis-associated factor 1 (XAF1) is a tumor suppressor that is commonly inactivated in multiple human cancers. However, its role in the pathogenesis and therapeutic response of glioma is poorly characterized.

**Methods:**

XAF1 activation by temozolomide (TMZ) and its effect on TMZ cytotoxicity were defined using luciferase reporter, flow cytometry, and immunofluorescence assays. Signaling mechanism was analyzed using genetic and pharmacologic experiments. *In vivo* studies were performed in mice to validate the role of XAF1 in TMZ therapy.

**Results:**

Epigenetic alteration of *XAF1* is frequent in cell lines and primary tumors and contributes to cancer cell growth. *XAF1* transcription is activated by TMZ via JNK–IRF-1 signaling to promote apoptosis while it is impaired by promoter hypermethylation. In tumor cells expressing high *O*^6^-methylguanine-DNA methyltransferase (MGMT), XAF1 response to TMZ is debilitated. XAF1 facilitates TMZ-mediated autophagic flux to direct an apoptotic transition of protective autophagy. Mechanistically, XAF1 is translocated into the mitochondria to stimulate reactive oxygen species (ROS) production and ataxia telangiectasia mutated (ATM)–AMP-activated protein kinase (AMPK) signaling. A mutant XAF1 lacking the zinc finger 6 domain fails to localize in the mitochondria and activate ROS–ATM–AMPK signaling and autophagy-mediated apoptosis. XAF1-restored xenograft tumors display a reduced growth rate and enhanced therapeutic response to TMZ, which is accompanied with activation of ATM–AMPK signaling. XAF1 expression is associated with overall survival of TMZ treatment patients, particularly with low MGMT cancer.

**Conclusions:**

This study uncovers an important role for the XAF1–ATM–AMPK axis as a linchpin to govern glioma response to TMZ therapy.

**Key Points**

- *XAF1* is activated by TMZ to evoke TMZ-induced apoptosis.
- XAF1 directs apoptotic switch of protective autophagy through ROS–ATM–AMPK signaling.
- XAF1–ATM–AMPK axis functions as a linchpin to govern the fate of TMZ-treated glioma cells.

**Importance of the Study**

XAF1 expression is commonly altered in GBMs. Here, we show that epigenetic alteration of *XAF1* or high expression of MGMT contributes to GBM resistance to TMZ therapy by debilitating *XAF1* induction by TMZ. XAF1 acts as a molecular linchpin to govern tumor response to TMZ favoring apoptosis over autophagy, identifying a new attractive target for therapeutic intervention of GBM.

Glioblastoma multiforme (GBM) is the most aggressive astrocytoma classified as grade IV astrocytoma.^1–3^ GBM rarely metastasizes to other organs but extensively infiltrates nearby normal brain parenchyma.^4^ Although accumulating evidence indicates the involvement of multiple pro-oncogenes and tumor suppressor genes, including isocitrate dehydrogenase-1 (IDH1), epidermal growth factor receptor (EGFR), and TP53, the underlying molecular events that drive the neoplastic process in GBM are largely undefined.^5^ GBM is currently incurable with a median survival of 12.2 months after patient diagnosis.^1–3^ Although postoperative radiotherapy concurrently with temozolomide (TMZ) have shown to increase overall survival, it only modestly improves survival, with recurrent tumors showing a strong drug-resistant phenotype.^6,7^

TMZ is an oral SN1 mono-alkylating agent that produces *O*^6^-methylguanine (*O*^6^-MeG) base lesions and its toxicity is mediated primarily by DNA mismatch repair (MMR)-dependent processing at *O*^6^-MeG lesions.^6–8^ During replication, *O*^6^-MeG mismatches with thymine and futile MMR cycling at *O*^6^-MeG lesions generate double-strand breaks (DSBs), resulting in cell-cycle arrest and/or cell death. This MMR processing can be prevented by *O*^6^-methylguanine-DNA methyltransferase (MGMT), which removes the methyl group from the *O*^6^ position of guanine.^9^ Suppression of MGMT expression leads to persistent *O*^6^-MeG lesions, thereby leading to collapsed replication forks and cell death.^10–12^

X-linked inhibitor of apoptosis (XIAP)-associated factor 1 (XAF1) is a pro-apoptotic tumor suppressor that is frequently inactivated in multiple human cancers mainly due to aberrant promoter CpG sites hypermethylation.^13–16^ Epigenetic inactivation of *XAF1* is associated with the stage and grade of many tumors, including castration-resistant prostate cancer, supporting its implication in the malignant progression of tumors.^13–17^ XAF1 is originally reported to trigger a redistribution of XIAP from the cytosol to the nucleus to evoke its pro-apoptotic effect.^13^ However, in certain tumor cells undergoing XAF1-driven apoptosis, the nuclear translocation of XIAP is not recognized, and XAF1 shows comparable apoptotic ability in *XIAP*^−/−^ and *XIAP*^+/+^ cells, indicating that XAF1 can induce apoptosis via XIAP-independent mechanisms.^16,18,19^

XAF1 enhances tumor cell sensitivity to various apoptotic stimuli, such as genotoxic, oxidative, and cytokine stresses and is involved in the regulation of autophagy and G2/M checkpoint of the cell cycle.^15,20–22^ Recently, we reported that XAF1 is activated by p53 and interferon regulatory factor (IRF)-1 and directs an apoptotic switch of p53′ cell-fate decisions function and destabilizes metallothionein 2A to drives cytotoxic metal stress response.^23–25^

In this study, we observed that XAF1 induction by TMZ directs an apoptotic switch of protective autophagy, identifying XAF1 as a critical tumor suppressor that governs the glioma response to TMZ therapy.

## Materials and Methods

### Tumor Tissues, Cell Lines, and Patient Database Analysis

Sixteen primary glioma tissues and 5 noncancerous brain tissues were obtained from Korea University Medical Center. Seven glioma cell lines (U87MG, A1207, LN18, LN229, T98G, U138MG, and U373MG) were obtained from American Type Culture Collection. For GBM patient database analysis, the Cancer Genome Atlas (TCGA) database and public RNA-seq data were utilized. Description of subline establishment and data mining is presented in Supplementary Methods.

### Semiquantitative Polymerase Chain Reaction, 5-Aza-dC Treatment, and Bisulfite DNA Sequencing Analysis

RT-PCR was repeated at least 3 times for each specimen, and the means were obtained. For the detection of sequence alterations, RT-PCR single-strand conformational polymorphism analysis was performed as described previously.^14^ Bisulfite DNA sequencing analysis for the *XAF1* promoter was performed as described previously.^16^ Detailed description of the assay is found in Supplementary Methods.

### Immunoblotting, Immunoprecipitation, and Immunohistochemistry

The protocols for immunoblotting and preparation of cytosolic, nuclear, and mitochondrial extracts have been described previously.^15,23^ Description of antibodies and immunohistochemistry assay is presented in Supplementary Methods.

### Transfection, Cell Growth, Luciferase Reporter, and Chromatin Immunoprecipitation Assays

XAF1 expression vectors used in this study were described previously.^23–25^ Cell proliferation and apoptosis were determined using cell number counting, trypan blue exclusion, and flow cytometry analysis. Cloning of the *XAF1* promoter and site-directed mutagenesis of IRF-1 binding site were described previously.^15,16,25^ Detailed description of assays is presented in Supplementary Methods.

### Reactive Oxygen Species Measurement and Mitochondria Membrane Permeability

Cells were incubated with 2′,7′-dichlorofluorescin diacetate (DCFH-DA) and reactive oxygen species (ROS) level was measured using microscopic and microplate analysis. The mitochondrial membrane potential (ΔΨm) was determined by using flow cytometric analysis of JC-1 (tetraethyl-benzimidazolylcarbo-cyanine iodide). Detailed description of assays is presented in Supplementary Methods.

### Immunofluorescence and Microscopic Autophagy Assays

Cells were transfected with expression vectors encoding a green fluorescent protein (GFP) and MitoTracker (Thermo Fisher Scientific) was used for the counterstaining of the mitochondria. For autophagy detection, U87MG cells were cotransfected with GFP-LC3B and either red fluorescent protein (RFP)-control or RFP-XAF1. Punctuated images of GFP-LC3 were obtained with the confocal microscope. Detailed description of assay is presented in Supplementary Methods.

### Animal Studies

Tumor xenograft assay was carried out as described.^23–25^ All studies were performed with the approval of Korea University Institutional Animal Care and Use Committee and Korea Animal Protection Law. Detailed description of assay is presented in Supplementary Methods.

### Statistical Analysis

All experiments, including cell number counting, reporter luciferase, flow cytometry, and colony-forming assays, were repeated 3 times, and the results were presented as mean values ± SD. Mean and SD values were calculated using Microsoft Excel software. A Student’s *t* test (GraphPad Prism8 software, CA) was performed to determine the statistical significance. One-way ANOVA with a post hoc test was used for comparing multiple independent variables. A *P* value of less than .05 was considered significant.

## Results

### Epigenetic Alteration of *XAF1* Contributes to Glioma Cell Growth and Resistance to TMZ

**Figure 1.**
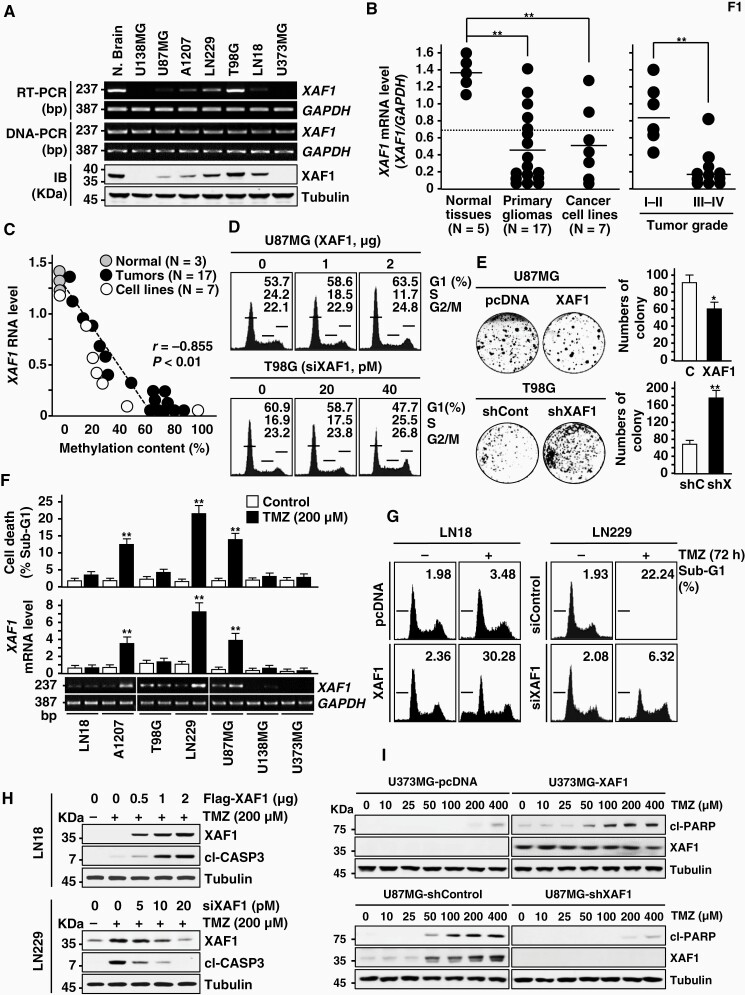
XAF1 expression in human glioma cell lines and its effect on cell response to TMZ. (A) Genomic and expression status of XAF1 were determined by DNA-PCR, RT-PCR, and immunoblot (IB). N, normal tissue. (B) Expression levels of *XAF1* mRNA in tissues and cell lines and its association with tumor grade. Bar indicates the mean mRNA levels of each group. Data are presented as mean ± SD of triplicate assays. Student’s *t* tests were performed (**P < .01). (C) Inverse correlation between *XAF1* mRNA levels and promoter methylation content. *r*, Pearson’s correlation coefficient. (D) XAF1 induction of G1 cell-cycle arrest. Cells were transfected with XAF1 expression vectors or siXAF1 molecules as indicated. After 48 h transfection, flow cytometric analysis of the cell-cycle phases was carried out. (E) XAF1 suppression of colony-forming ability of tumor cells. Cells were maintained for 10 days and colonies were stained with crystal violet. Data are presented as mean ± SD of triplicate assays (*P < .05; **P < .01). (F) TMZ upregulation of *XAF1* and its association with apoptosis induction. Apoptosis was determined by flow cytometric analysis of sub-G1 fraction. Data are presented as mean ± SD of triplicate assays (**P < .01). (G–I) Effect of XAF1 overexpression and knockdown on tumor cell response to TMZ cytotoxicity. LN18 and LN229 were transfected with Flag-XAF1 (2 µg/mL) and siXAF1 (50 pM), respectively, and treated with TMZ (200 mM). U373MG sublines (pcDNA and XAF1) and U87MG sublines (shControl and shXAF1) were exposed to an increasing dose of TMZ for 72 h. Apoptosis induction was determined by flow cytometric analysis of sub-G1 fraction and IB assay of cleaved caspase-3 (cl-CASP3) and cl-PARP levels. TMZ, temozolomide.

To explore the candidacy of XAF1 as a suppressor in glioma pathogenesis, we initially examined the expression status of XAF1 in cell lines and tissues. Five of 7 (71.4%) cell lines and 12 of 17 (70.6%) primary tumors were observed to have *XAF1* mRNA levels less than a half (0.69) of normal means (1.38) (Figure 1A and B; Supplementary Figure S1A). Abnormal reduction of *XAF1* mRNA (<0.69) was more common in high-grade tumors (III–IV, 10 of 11 [91%]) than low grade tumors (I–II, 3 of 6 [50%]) (Figure 1B). Immunoblot and immunohistochemical assays of the same cell lines and tissues showed that protein levels are consistent with mRNA levels (Supplementary Figure S1B). While none of the cell lines and tissues exhibited detectable reduction of *XAF1* gene level, all low-expressor cells showed mRNA induction following treatment with the demethylating agent 5-Aza-dC (Supplementary Figure S1C). Therefore, we determined the methylation status of 8 CpG sites, including a CpG site due to single nucleotide polymorphism (rs3785793) within the 5′ proximal region of the *XAF1* promoter (Supplementary Figure S1D and E). A bisulfite DNA sequencing analysis revealed that the overall methylation frequency correlates tightly with mRNA level (Figure 1C; Supplementary Figure S1F–I). On this basis, we examined XAF1 effect on cell growth and observed that the G1 to S transition of the cell cycle and colony-forming ability are inhibited by XAF1 expression (Figure 1D and E; Supplementary Figure S1J and K).

Next, we asked whether XAF1 expression is affected by TMZ. *XAF1* mRNA is upregulated by TMZ in 3 of 7 cell lines (A1207, LN229, and U87MG) with relatively low promoter methylation and its induction is linked to apoptotic response to TMZ while 2 cell lines (U138MG and U373MG) with high promoter methylation failed to induce both *XAF1* and apoptosis, supporting that XAF1 induction by TMZ is hindered by aberrant promoter hypermethylation (Figure 1F; Supplementary Figure S1L and M). Meanwhile, no induction of *XAF1* in 2 low methylation cell lines (LN18 and T98G) indicates that TMZ induction of *XAF1* is inhibited by other mechanism(s) in these cells. Apoptotic response to TMZ was markedly up- and downregulated by overexpression and knockdown of XAF1, respectively (Figure 1G and H; Supplementary Figure S1N). Likewise, U373MG-XAF1 and U87MG-shXAF1 sublines showed enhanced and reduced apoptotic sensitivity to TMZ compared to U87MG-pcDNA and T98G-shXAF1 sublines, respectively (Figure 1I). Three different siRNAs against XAF1 showed comparable effect on TMZ-induced cell death, excluding the off-target effect of siRNA (Supplementary Figure S1O).

### XAF1-Mediated TMZ Cytotoxicity Is Attenuated by MGMT

**Figure 2.**
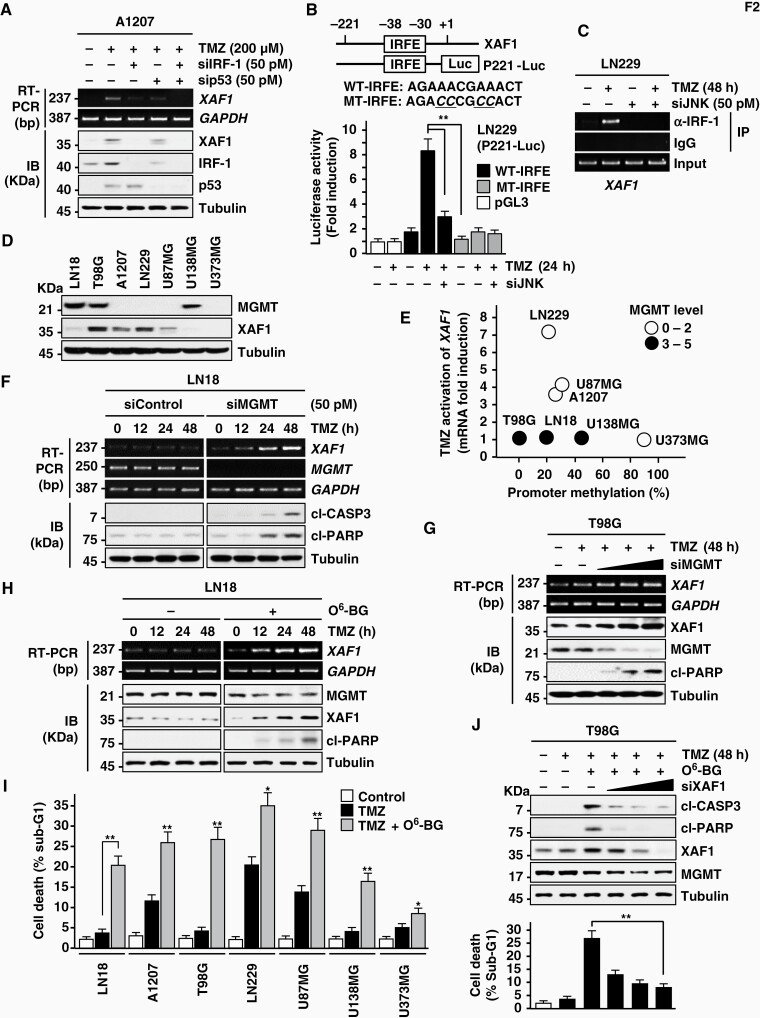
MGMT effect on TMZ induction of XAF1. (A) Disruption of XAF1 induction by depletion of IRF-1 or p53. Transfected cells were exposed to TMZ for 24 h. (B) Reporter construction and luciferase assay. Effect of JNK depletion or IRFE mutation on the reporter responsiveness to TMZ (200 mM) was determined. (C) Effect of JNK depletion on IRF-1 binding to the IRFE within the *XAF1* promoter. Cells were transfected with siJNK and then exposed to TMZ (200 mM) for 48 h. IRF-1 binding to the IRFE was determined by a ChIP assay using an IRF-1-specific antibody and PCR amplification of the promoter region. (D) IB assay showing expression status of MGMT in glioma cell lines. (E) TMZ activation of *XAF1* mRNA and its association with *XAF1* promoter methylation and MGMT expression in cell lines. (F, G) Effect of MGMT depletion on TMZ induction of XAF1. LN18 and T98G cells were transfected with siMGMT and exposed to TMZ (200 mM) as indicated. (H) Effect of *O*^6^-BG pretreatment on TMZ induction of XAF1. Cells were incubated with *O*^6^-BG (20 mM) for 2 h before TMZ treatment (200 nM). (I) Effect of *O*^6^-BG on TMZ-induced apoptosis in glioma cell lines. Cells were preincubated with *O*^6^-BG (20 mM, 2 h) and then exposed to TMZ (200 mM, 72 h). Data are presented as mean ± SD of triplicate assays. Student’s *t* tests were performed (*P < .05; **P < .01). (J) Effect of XAF1 depletion on *O*^6^-BG enhancement of TMZ cytotoxicity. T98G cells were transfected with an increasing dose of siXAF1. The cells were preincubated with *O*^6^-BG (20 mM, 2 h) before exposure to TMZ (200 mM). Data are presented as mean ± SD of triplicate assays (**P < .01). ChIP, chromatin immunoprecipitation; MGMT, *O*^6^-methylguanine-DNA methyltransferase; TMZ, temozolomide.

We have shown that stress induction of *XAF1* transcription occurs through the p53 and JNK–IRF-1 signaling pathways.^23,25^ Consistently, we identified that *XAF1* induction is attenuated partially by depletion of either p53 or IRF-1 and completely blocked by their codepletion (Figure 2A). In LN229 cells harboring mutant p53, *XAF1* induction was disrupted by knockdown of either JNK or IRF-1 while it is not affected by p38 and STAT3 (Supplementary Figure S2A). A luciferase assay using the Pro/221-Luc construct comprising the IRF element (IRFE) showed that TMZ activation of the reporter is disrupted if the IRFE is mutated (MT-IRFE) or JNK is depleted (Figure 2B). A chromatin immunoprecipitation assay revealed that IRF-1 binds to the IRFE and this binding is abolished by JNK depletion (Figure 2C).

TMZ toxicity is primarily linked to *O*^6^-MeG production and glioma response to TMZ therapy is inversely associated with expression of MGMT, a key protein responsible for the repair of *O*^6^-MeG base lesions.^6–9^ We asked whether MGMT attenuates XAF1 response to TMZ. An immunoblot assay showed high expression of MGMT in 3 cell lines (T98G, LN18, and U138MG) and low or negligible expression in the other 5 cell lines (Figure 2D). Among 4 cell lines with no XAF1 response to TMZ, 3 cell lines (T98G, LN18, and U138MG) were identified to have high expression of MGMT while the other (U373MG) has aberrantly promoter hypermethylation (Figures 2E and 1F). U138MG was shown to have high MGMT as well as relatively high promoter methylation. This supports that TMZ-mediated *XAF1* upregulation is influenced by promoter methylation and MGMT expression. As predicted, in both T98G and LN18 cells, TMZ activation of *XAF1* and its cytotoxicity were restored by either siRNA-mediated MGMT depletion or pretreatment with the MGMT inhibitor *O*^6^-BG (Figure 2F–I; Supplementary Figure S2B and C). The enhanced TMZ cytotoxicity by *O*^6^-BG treatment was not detected if XAF1 induction was inhibited (Figure 2J). MGMT expression was not affected by XAF1 (Supplementary Figure S2D). Unlike TMZ, genotoxic agents, such as etoposide, cisplatin, H_2_O_2_, and γ-irradiation caused a clear induction of *XAF1* mRNA expression in T98G cells, supporting the TMZ specificity of MGMT action (Supplementary Figure S2E–G).

### XAF1 Evokes TMZ Cytotoxicity Through Activation of ATM–AMPK Signaling

**Figure 3.**
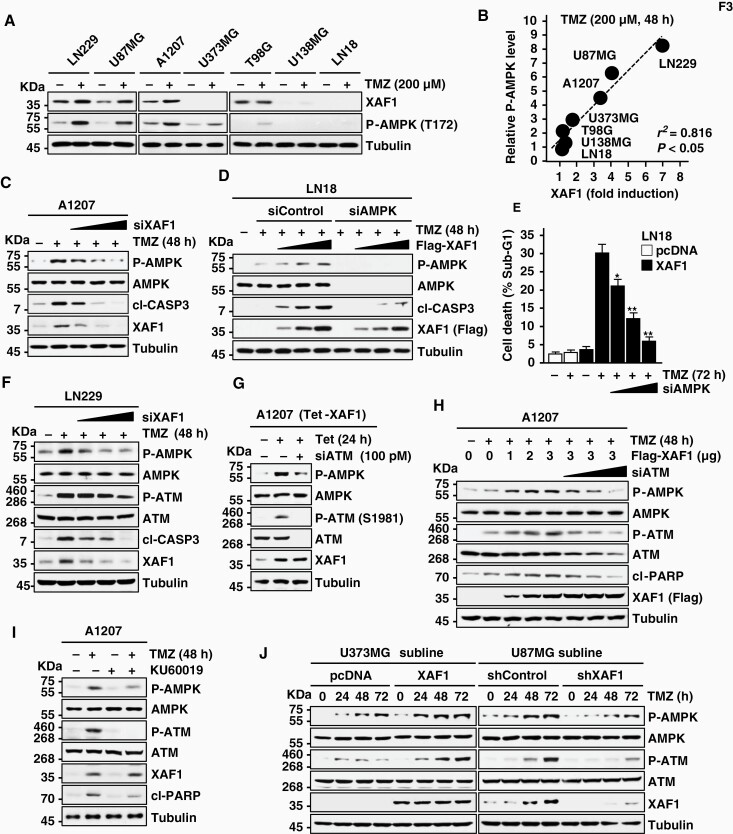
XAF1 activation of ATM–AMPK signaling. (A, B) XAF1 induction by TMZ and its association with AMPK phosphorylation (P-AMPK). Cells were exposed to TMZ (200 nM, 48 h) and IB assay was performed to determine XAF1 and P-AMPK expression. *r*, Pearson’s correlation coefficient. (C) Effect of XAF1 depletion on TMZ induction of P-AMPK. (D, E) Effect of AMPK depletion on XAF1 enhancement of TMZ cytotoxicity. LN18 cells were transfected as indicated and treated with TMZ (200 µM). Data are presented as mean ± SD of triplicate assays (*P < .05; **P < 0.01). (F) Effect of XAF1 depletion on TMZ-induced ATM phosphorylation (P-ATM). LN229 cells were transfected with an increasing dose of siXAF1 and then treated with TMZ (200 mM, 48 h). (G, H) Effect of ATM depletion on XAF1-induced AMPK phosphorylation. A1207 (Tet-XAF1) cells were transfected with siATM (100 pM). After 48 h, the cells were exposed to tetracycline (Tet, 10 µg/mL) to activate XAF1 expression. (I) Effect of ATM inhibitor on TMZ activation of AMPK. A1207 cells were incubated with KU60019 (10 µM, 2 h) before TMZ treatment (200 µM, 48 h). (J) Effect of XAF1 overexpression and knockdown on TMZ activation of ATM and AMPK. U373MG sublines (pcDNA and XAF1) and U87MG sublines (shControl and shXAF1) were exposed to TMZ (200 nM) for the indicated times. AMPK, AMP-activated protein kinase; ATM, ataxia telangiectasia mutated; IB, immunoblot; TMZ, temozolomide.

XAF1 is originally reported to induce the nuclear sequestration of XIAP to exert its apoptosis-promoting effect.^13^ However, we observed that XAF1 stimulation of TMZ-induced apoptosis is not affected by XIAP depletion and that XAF1 does not trigger the subcellular redistribution of XIAP (Supplementary Figure S3A–C). We assessed the involvement of AMPK, which is known to play a regulatory role in tumor cell response to TMZ.^26^ In response to TMZ, 7 cell lines displayed variable induction of AMPK phosphorylation (P-AMPK) that is associated with XAF1 induction (Figure 3A and B). TMZ induction of P-AMPK was down- and upregulated by depletion and expression of XAF1, respectively (Figure 3C and D). XAF1’s pro-apoptotic effect was impaired by AMPK depletion (Figure 3D and E; Supplementary Figure S3D and E). XAF1 induction of P-AMPK was also detected in multiple nonglioma cells, including IMR90 (fibroblast), T47D (breast), HCT116 (colon), and U2OS (sarcoma) and XAF1 depletion disrupted TMZ cytotoxicity in XAF1 expression subline of PC3 (prostate) (Supplementary Figure S3F–H). A series of assay revealed that XAF1 does not interact with AMPK and not regulate expression of AMPK subunits (α, β, and γ) and that XAF1 induction of P-AMPK is not affected by depletion of 3 representative AMPK upstream kinases (LKB1, CAMKKβ, and TAK1) (Supplementary Figure S3I–L). In contrast, XAF1 increased P-ATM (S1981), and XAF1 induction of P-AMPK was profoundly impeded by either depletion of ATM or pretreatment of the ATM inhibitor KU60019 (Figure 3F–I). Compared to controls, U373MG-XAF1 and U87MG-shXAF1 sublines showed much higher and lower P-ATM, respectively, following TMZ treatment (Figure 3J).

### XAF1 Induces P-ATM Through Mitochondrial ROS Production

**Figure 4.**
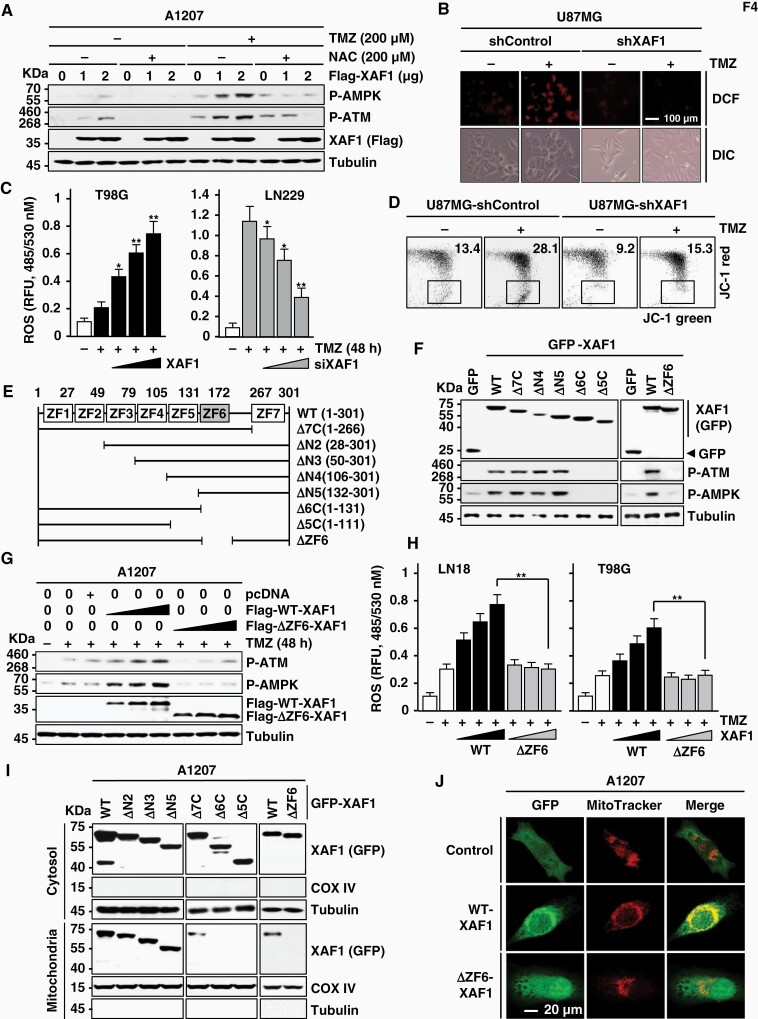
Activation of ROS–ATM–AMPK signaling by mitochondrial XAF1. (A) IB assay showing ROS dependency of XAF1 activation of ATM and AMPK. Cells were transfected with Flag-XAF1 as indicated and exposed to TMZ (48 h). The reducing reagent NAC was added to cells 2 h before TMZ treatment. (B) XAF1 enhancement of ROS production. U87MG sublines (shControl and shXAF1) were exposed to TMZ (200 µM) for 48 h. The cells were incubated with 2′,7′-dichlorofluorescin diacetate (DCFH-DA, 10 µM) for 30 min and ROS production was detected using a fluorescence microscope. DIC, differential interface contrast. (C) Effect of XAF1 overexpression and depletion on TMZ-induced ROS production. Cells were transfected with increasing doses of XAF1 and siXAF1 as indicated and then treated with TMZ (200 µM, 48 h). The cells were incubated with DCFH-DA (20 µM, 30 min). Intracellular ROS level was monitored by measuring the fluorescence intensity of cells. Data are presented as mean ± SD of triplicate assays (*P < .05; **P < .01). (D) JC-1 fluorescence assay showing XAF1 effect on mitochondria membrane permeability. Cells treated with TMZ (200 µM, 72 h) were incubated with JC-1 (250 nM). (E) Construction of XAF1 deletion mutants. ZF, zinc finger. (F) Identification of ZF6 as an essential region of XAF1 for ATM–AMPK activation. IB assay was performed using T98G cells to compare effect of GFP-XAF1 constructs on P-ATM and P-AMPK levels. (G, H) Comparison of WT- and ΔZF6-XAF1 activity to induce ATM–AMPK signaling and ROS production. Transfected cells were exposed to TMZ (200 µM, 48 h). Data are presented as mean ± SD of triplicate assays (**P < .01). (I, J) IB and immunofluorescence assays of XAF1 showing a critical role for ZF6 in the mitochondrial translocation of XAF1. A1207 cells were transfected with XAF1 constructs as indicated and IB assays were performed using the cytosolic and mitochondrial fractions of the cells. COX IV and Tubulin were used as a marker for the mitochondrial and cytosol fractions, respectively. For immunofluorescence assay, A1207 cells were transfected with either GFP-WT-XAF1 or GFP-∆ZF6-XAF1. MitoTracker was used to visualize the mitochondria. AMPK, AMP-activated protein kinase; ATM, ataxia telangiectasia mutated; IB, immunoblot; NAC, *N*-acetyl-cysteine; ROS, reactive oxygen species; TMZ, temozolomide.

Given that ATM autophosphorylation is triggered by ROS, we tested whether XAF1 activates ATM via ROS generation.^27^ XAF1 induction of P-ATM was strongly suppressed by the ROS blocker *N*-acetyl-cysteine (Figure 4A; Supplementary Figure S4A and B). ROS production by TMZ was much lower in U87MG-shXAF1 versus U87MG-shControl and up- and downregulated by XAF1 expression and depletion, respectively (Figure 4B and C; Supplementary Figure S4C and D). Next we tested whether XAF1 triggers mitochondrial ROS generation. To address this issue, we examined XAF1 effect on the mitochondria membrane permeability (MMP). U87MG-shXAF1 cells displayed substantially reduced MMP compared to U87MG-shControl in both the presence and absence of TMZ treatment (Figure 4D). An immunoprecipitation assay using a series of deletion constructs, we identified that the zinc finger (ZF) 6 domain plays a key role in XAF1 activation of ATM and AMPK (Figure 4E–G). A mutant lacking the ZF6 domain (ΔZF6-XAF1) showed no activity to stimulate ROS production and apoptosis induction (Figure 4H; Supplementary Figure S4E). Furthermore, a series of assays using cell fractionations and immunofluorescence detected the mitochondrial distribution of WT-XAF1 but not of ΔZF6-XAF1, indicating that the ZF6 domain is essential for the mitochondrial localization of XAF1 (Figure 4I and J). The nuclear localization of XAF1 was not affected by the ZF6 deletion (Supplementary Figure S4F). These support that mitochondrial XAF1 triggers TMZ cytotoxicity through activation of ROS–ATM–AMPK signaling.

### XAF1 Induces Apoptotic Switch of Protective Autophagy

**Figure 5.**
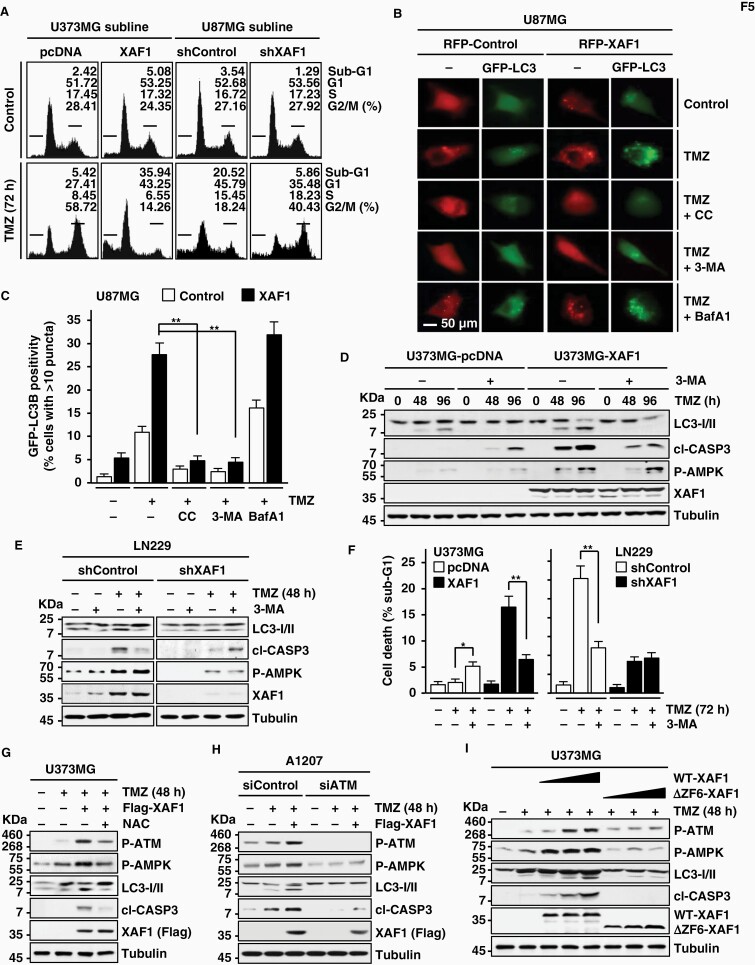
XAF1 induction of autophagy-mediated apoptosis. (A) Effect of XAF1 expression on glioma cell response to TMZ. Cells were treated with TMZ (200 µM) and cell-cycle progression was determined using flow cytometry. (B, C) Immunofluorescence assay showing XAF1 stimulation of autophagic flux. Cells were co-transfected with GFP-LC3 and RFP-XAF1 as indicated and exposed to Compound C (CC, 10 µM), 3-MA (5 µM), or BafA1 (100 nM) for 2 h before TMZ treatment (200 µM, 48 h). Percentages of cells showing GFP-LC3 puncta were calculated. Data are presented as mean ± SD of triplicate assays (**P < .01). (D–F) IB and flow cytometry assays showing an apoptotic switch of protective autophagy by XAF1. Cells were exposed to 3-MA (5 µM, 2 h) before TMZ treatment (200 µM). Data are presented as mean ± SD of triplicate assays (*P < .05; **P < .01). (G, H) Effect of ROS blockade or ATM depletion on XAF1 induction of autophagy-mediated apoptosis. Cells were transfected with Flag-XAF1 and/or siATM as indicated and incubated with NAC (200 µM, 2 h) before TMZ treatment (200 µM). (I) Comparison of WT- and ΔZF6-XAF1 ability to induce autophagy-mediated apoptosis. Transfected cells were treated with TMZ (200 µM). ATM, ataxia telangiectasia mutated; IB, immunoblot; NAC, *N*-acetyl-cysteine; ROS, reactive oxygen species; TMZ, temozolomide.

TMZ-induced cell-cycle arrest is linked to protective autophagy.^28–30^ We thus asked whether XAF1 affects TMZ-induced cell-cycle arrest and autophagy. Upon exposure to TMZ, U373MG-pcDNA manifested cytostatic response (G2/M cell-cycle arrest) while U373MG-XAF1 showed cytotoxic response (apoptosis induction) (Figure 5A). Likewise, U87MG-pcDNA exhibited apoptotic response while U87MG-shXAF1 showed G2/M arrest response, supporting that XAF1 induces a cytostatic to cytotoxic switch of TMZ effect. Moreover, an immunofluorescence assay revealed that XAF1 increases TMZ-induced LC3B positivity and this effect is suppressed by the AMPK inhibitor Compound C (CC), indicating that XAF1 promotes TMZ-induced autophagy through AMPK activation (Figure 5B and C). XAF1 induction of LC3B puncta was also observed in LN18 and U373MG and other types of cells, such as T47D (breast) and AGS (stomach) (Supplementary Figure S5A). To clarify whether the XAF1 induction of LC3B puncta reflects autophagy activation or results from its accumulation due to blockade of autophagic flux, we compared effect of 3-MA (inhibitor of early autophagy) and BafA1 (inhibitor of late autophagy). XAF1 induction of LC3B puncta was strongly suppressed by 3-MA but further increased by BafA1, indicating that XAF1 facilitates autophagic flux (Figure 5B and C). XAF1 induction of autophagy was also observed in glucose-deprived U87MG cells (Supplementary Figure S5B). Furthermore, compared to U373MG-pcDNA, U373MG-XAF1 exhibited higher sensitivity to TMZ-induced autophagy and apoptosis, both of which were decreased by 3-MA pretreatment (Figure 5D and F). A consistent result was observed from assays using LN229-shXAF1 and LN229-shControl (Figure 5E and F). A cell viability assay using U87MG sublines also revealed that XAF1-induced cell death is blocked by 3-MA (Supplementary Figure S5C). In contrast, 3-MA treatment led to apoptosis elevation in U373MG-pcDNA and LN229-shXAF1 cells, indicating that TMZ-triggered autophagy plays a protective role under XAF1-depleted conditions (Figure 5D–F; Supplementary Figure S5D). As predicted, XAF1 induction of autophagy is inhibited by either ROS blockade or ATM depletion while it is facilitated by WT-XAF1 but not affected by ΔZF6-XAF1 (Figure 5G–I). XAF1 was also identified to activate AMPK-ULK1-Raptor autophagy signaling and its function to increase autophagy and apoptosis is impeded by ULK1 depletion (Supplementary Figure S5E and F). These indicate that XAF1 drives TMZ cytotoxicity through activation of autophagy-mediated apoptosis.

### XAF1 Enhances Glioma Response to TMZ In Vivo

**Figure 6.**
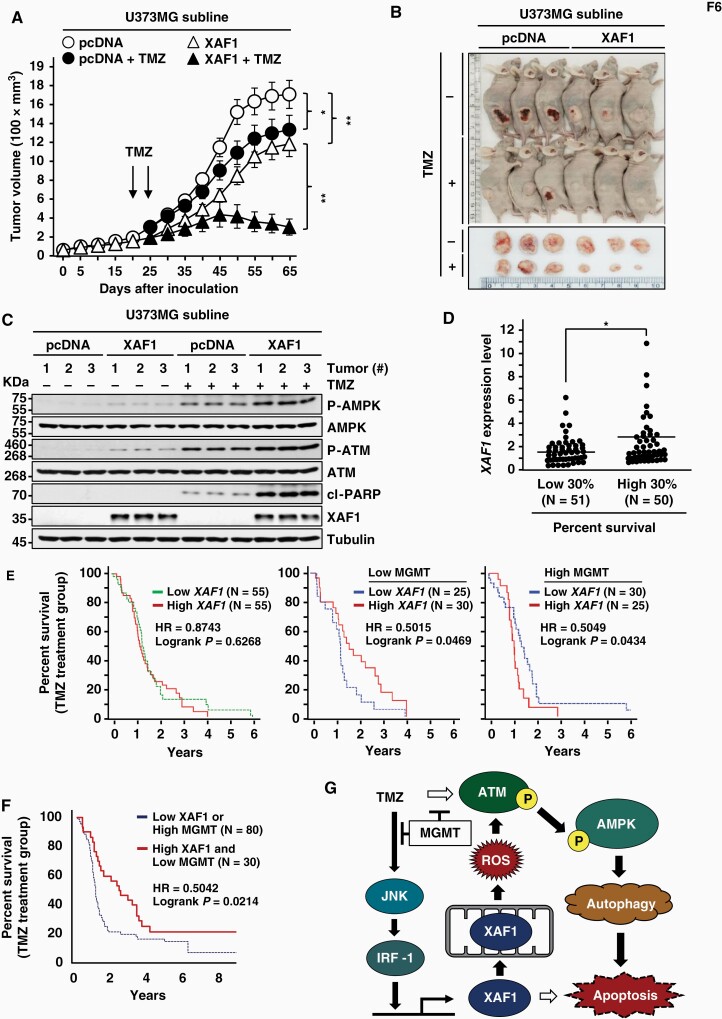
XAF1 mediates TMZ-induced tumor regression. (A) Mouse tumor xenograft assay showing XAF1 effect on tumor growth and response to TMZ. Six mice were used per group and TMZ (40 mg/kg) was injected twice as indicated. Data are presented as mean ± SD (*P < .05; **P < .01). (B) Representative photographs of xenograft tumors at day 65 after inoculation. (C) IB assay of P-AMPK, P-ATM, and cl-PARP levels in xenograft tumor tissues. (D) TCGA data analysis showing XAF1 association with overall survival of GBM patients. *XAF1* mRNA level was compared between high and low 30% of survival groups. Data are presented as mean ± SD (*P < .05). (E, F) XAF1 association with the survival of TMZ treatment patients with high and low MGMT levels. (G) Schematic representation of XAF1 activation by TMZ and XAF1 induction of autophagy-mediated apoptosis through ROS–ATM–AMPK signaling. AMPK, AMP-activated protein kinase; ATM, ataxia telangiectasia mutated; GBM, glioblastoma multiforme; IB, immunoblot; MGMT, *O*^6^-methylguanine-DNA methyltransferase; ROS, reactive oxygen species; TCGA, The Cancer Genome Atlas; TMZ, temozolomide.

To delineate the role for XAF1 in TMZ therapy, we carried out tumor xenograft assays using U373MG sublines. As predicted, U373MG-XAF1 tumors displayed lower growth rate compared to U373MG-pcDNA tumors (Figure 6A and B). Following TMZ injection, U373MG-XAF1 tumors exhibited significantly higher regression compared to U373MG-pcDNA tumors (80% vs 24%). U373MG-XAF1 tumors showed higher induction of P-ATM, P-AMPK, and cl-PARP following TMZ injection compared to U373MG-pcDNA tumors (Figure 6C). Moreover, mice bearing U373MG-XAF1 tumors showed higher survival compared to mice bearing U373MG-pcDNA tumors (Supplementary Figure S6A). Finally, to elicit the clinical relevance of XAF1, we performed targeted prognostic analysis using the TCGA GBM patient database. A comparative analysis of high and low survival groups revealed that *XAF1* mRNA level is associated with overall survival of patients (Figure 6D). Next, we asked whether *XAF1* expression is related to survival of TMZ-treated patients and identified that *XAF1* expression is associated with overall survival of patients with low MGMT expression (Figure 6E). Moreover, TMZ-treated patients with both high XAF1 and low MGMT showed a significantly higher survival compared to patients with either low XAF1 or high MGMT (Figure 6F). Collectively, our study demonstrates that XAF1 plays a crucial role in GBM response to TMZ therapy by directing the apoptotic switch of protective autophagy through ROS–ATM–AMPK signaling (Figure 6G).

## Discussion

Epigenetic inactivation of *XAF1* due to aberrant promoter CpG sites hypermethylation was reported in various human cancers.^14–16^ A recent study of high-grade (III–IV) gliomas revealed that methylation of 3 CpG sites in the *XAF1* promoter is associated with *IDH1* status and improved clinical outcome.^31^ In the present study, we found that epigenetic inactivation of *XAF1* is highly frequent in glioma and that the promoter methylation content is associated with advanced tumor grade, cell growth, and chemoresistance to TMZ, indicating that XAF1 status could be a useful marker for the therapeutic efficacy of TMZ. Moreover, *XAF1* transcription is activated by TMZ and its induction is a critical event responsible for glioma response to TMZ therapy. We also found that XAF1 induction by TMZ occurs through JNK–IRF-1 and p53 signalings and drives TMZ cytotoxicity in the XIAP-independent manner. Intriguingly, a substantial fraction of XAF1 is localized in the mitochondria and the ZF6 domain is essential for its mitochondrial translocation and subsequent activation of ROS–ATM–AMPK signaling. This finding thus warrants further molecular studies for the mechanism underlying mitochondrial XAF1-mediated cell-fate decisions under various stressful conditions.

*MGMT* is downregulated by promoter methylation in almost half of GBM specimens and its inactivation is associated with enhanced tumor cell sensitivity and the patient response to TMZ.^10–12,32,33^ This raises the issue that MGMT-negative tumors require other chemoresistance mechanisms for TMZ tolerance. We found that MGMT attenuates TMZ induction of *XAF1* and XAF1-driven TMZ cytotoxicity is restored by blockade of MGMT, suggesting that persistent *O*^6^-MeG lesions due to MGMT inactivation may confer *XAF1* promoter response to TMZ, thereby accelerating TMZ cytotoxicity. This is supported by our database analysis showing that *XAF1* expression is associated with overall survival of TMZ treatment patients with low MGMT level. In this context, it will be valuable to characterize the methylation or expression status of *XAF1* in recurrent tumors with TMZ-resistant phenotype to understand the molecular mechanisms of therapeutic escape and to develop more effective treatment strategies against GBM.

Autophagy is a highly conserved catabolic process by which cells direct their own cytoplasmic components and organelles to lysosomes for degradation to maintain organelle function and central carbon metabolism for stress adaptation, homeostasis, and cell survival.^34–36^ However, in certain circumstances, autophagy leads to cell death, indicating that autophagy pathway can be subverted from a survival to a death function.^37,38^ Recent guidelines define the roles of autophagy in cell death as: (1) autophagy-associated cell death, where autophagy does not have an active role in it; (2) autophagy-mediated cell death, where autophagy triggers apoptosis; and (3) autophagy-dependent cell death, where autophagy induces a distinct cell death independently of apoptosis or necrosis.^39^ Although XAF1 was shown to induce autophagy in certain cancer cells, the molecular mechanism underlying XAF1 induction of autophagy and its linkage to XAF1’s pro-apoptotic function remain largely undefined.^20^ In this study, we identified that XAF1-mediated TMZ cytotoxicity stems from its autophagy-stimulating activity. We observed that XAF1-induced apoptosis is prevented if autophagy is blocked while autophagy inhibition increases apoptosis in XAF1-silenced or -depleted cells, indicating that XAF1 directs an apoptotic switch of TMZ-triggered protective autophagy to promote autophagy-mediated apoptosis.

TMZ induces G2/M arrest and abrogation of G2/M arrest potentiates TMZ-induced toxicity.^40,41^ TMZ induction of G2/M arrest occurs in a p53-independent manner while the duration of G2/M arrest is affected by p53 status.^42^ It was thus suggested that inhibition of the G2/M arrest pathway may represent a novel, mechanism-based means of increasing TMZ efficacy.^28,29,38^ However, the molecular mechanism by which TMZ response is converted from G2/M arrest to apoptosis remains undefined. We observed that TMZ induces G2/M arrest in both p53-proficient and -deficient tumor cells, and this G2/M arrest is linked to protective autophagy. Intriguingly, however, G2/M arrest-linked protective autophagy was subverted to apoptosis if XAF1 expression is activated while blockade of XAF1 induction leads to an apoptosis to autophagy switch. This finding indicates that XAF1 plays a key role in the ultimate fate decisions (survival or death) of TMZ-exposed glioma cells, further supporting the usefulness of XAF1 as a prognostic and therapeutic marker for the development of better treatment strategy.

AMPK is a well-documented kinase that is implicated in the regulation of cell-cycle arrest, autophagy, and apoptosis.^42^ Among several upstream kinases, such as LKB1, CaMKK, and TAK1, LKB1 is ubiquitously expressed and responsible for AMPK activation in most scenarios.^43^ In TMZ-exposed glioma cells, AMPK is activated by LKB1 to induce apoptosis and also activated by ATM to elicit protective autophagy.^26,43^ It is also known that increased ROS and/or ATM activation can directly activate AMPK.^44,45^ The ATM kinase is activated by DNA DSBs and orchestrates signaling cascades for the DNA damage response.^46^ The *O*^6^-MeG base lesions produced by TMZ can lead to DNA DSBs through subsequent futile cycles of DNA MMR.^6–9^ In this study, we found that AMPK activation by TMZ or XAF1 is not affected by depletion of LKB1, CaMKK, and TAK1. Meanwhile, we observed that ATM is activated by TMZ in both resistant and sensitive tumor cells, and XAF1 induction causes further activation of ATM, which is required for TMZ-induced apoptosis. It is thus conceivable that XAF1 activation leads to a conversion of ATM function favoring apoptosis over DNA repair. Moreover, XAF1 leads to ATM activation even in the absence of TMZ, suggesting that XAF1 may activate ATM through the DNA repair-independent signaling. ATM is activated by its oxidation in the absence of DNA damage, supporting that ATM functions as a sensor of ROS.^27^ Interestingly, our study shows that XAF1 enhances ROS production and a mitochondrial translocation-deficient mutant XAF1 fails to activate ROS–ATM–AMPK signaling and autophagy-mediated apoptosis. Further molecular studies will be required to address the mechanistic basis for the XAF1-induced mitochondrial dysfunction and ROS generation.

Collectively, this study indicates that XAF1 plays a key role in TMZ cytotoxicity by directing apoptotic switch of protective autophagy through ROS–ATM–AMPK signaling, illuminating the mechanistic consequence of epigenetic inactivation of *XAF1* in glioma pathogenesis.

## Supplementary Material

vdac013_suppl_Supplementary_Figures

vdac013_suppl_Supplementary_Materials

## Notes

**Authorship Statement**. M.-G.L., J.-S.C., N.-J.L., J.-S.L., K.-W.L., and K.-P.K. performed experiments. S.-H.K. collected and analyzed clinical samples. B.-G.R., S.-H.K., and S.-G.C. provided interpretation of the data. M.-G.L. and S.-G.C. wrote the manuscript. S.-G.C. is responsible for the designing and funding collections. All authors have approved this manuscript.
