# Supplementary material for: XAF1 directs glioma response to temozolomide through apoptotic transition of autophagy by activation of ATM–AMPK signaling
Source: Neurooncol Adv. 2022 Feb 7;4(1):vdac013. doi: 10.1093/noajnl/vdac013 (PMC8903238; doi:10.1093/noajnl/vdac013)
Supplement: vdac013_suppl_Supplementary_Figures [file vdac013_suppl_supplementary_figures.pptx]

## Slide 1
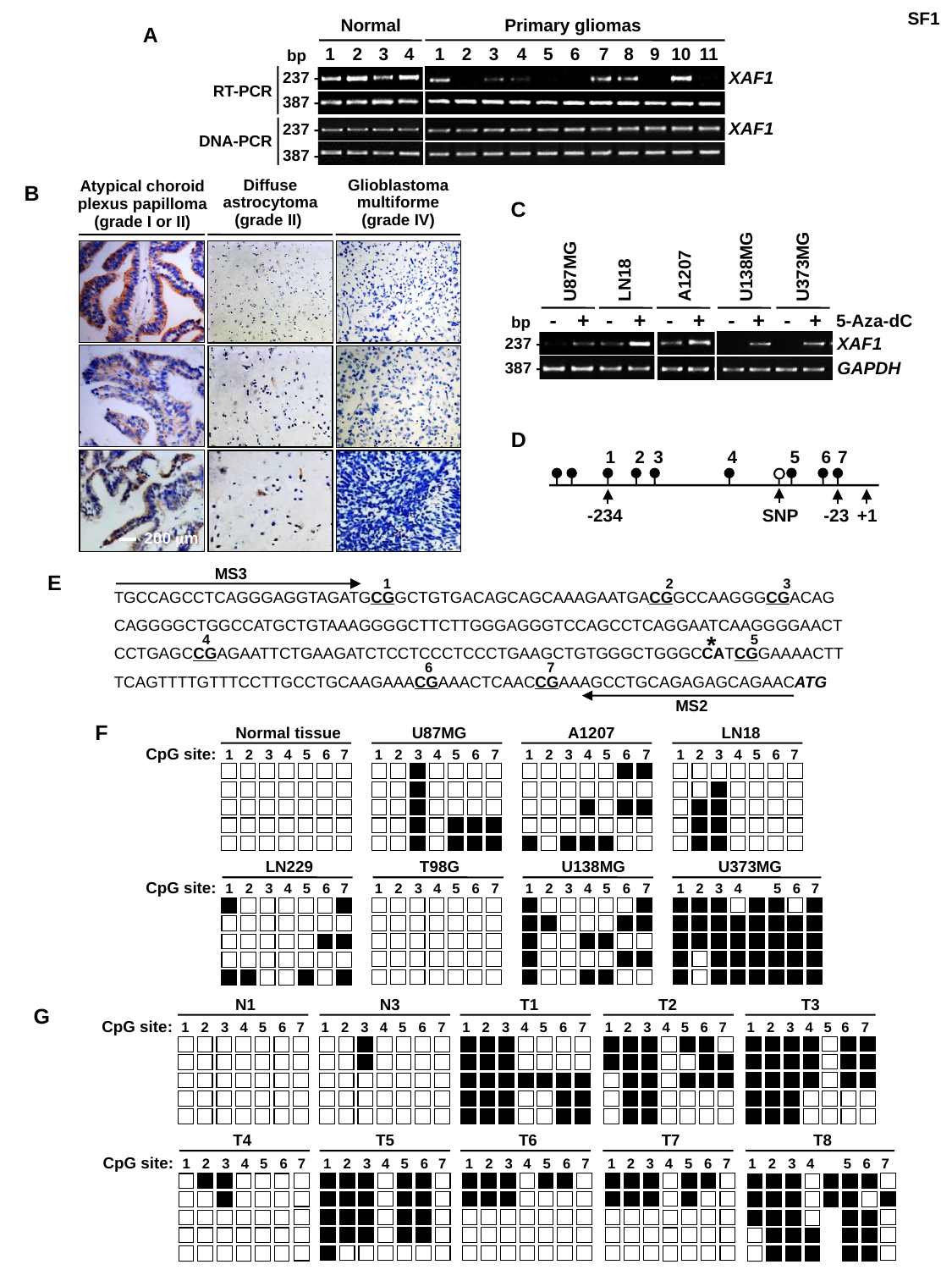

SF1
Normal Primary gliomas
1 2 3 4 1 2 3 4 5 6 7 8 9 10 11
bp
XAF1
GAPDH
237 -
387 -
237 -
387 -
RT-PCR
DNA-PCR
XAF1
GAPDH
A
Diffuse
astrocytoma
(grade II)
Glioblastoma
multiforme
(grade IV)
Atypical choroid
plexus papilloma
(grade I or II)
200 μm
B
C
U87MG
LN18
A1207
U138MG
U373MG
- + - + - + - + - +
5-Aza-dC
bp
237 -
387 -
XAF1
GAPDH
D
1 2 3 4 5 6 7
-234 SNP -23 +1
 (A/G)
MS3
 1 2 3 TGCCAGCCTCAGGGAGGTAGATGCGGCTGTGACAGCAGCAAAGAATGACGGCCAAGGGCGACAG
CAGGGGCTGGCCATGCTGTAAAGGGGCTTCTTGGGAGGGTCCAGCCTCAGGAATCAAGGGGAACT
 4 5
CCTGAGCCGAGAATTCTGAAGATCTCCTCCCTCCCTGAAGCTGTGGGCTGGGCCATCGGAAAACTT
 6 7
TCAGTTTTGTTTCCTTGCCTGCAAGAAACGAAACTCAACCGAAAGCCTGCAGAGAGCAGAACATG
*
MS2
E
F
Normal tissue U87MG A1207 LN18
CpG site: 1 2 3 4 5 6 7 1 2 3 4 5 6 7 1 2 3 4 5 6 7 1 2 3 4 5 6 7
LN229 T98G U138MG U373MG
CpG site: 1 2 3 4 5 6 7 1 2 3 4 5 6 7 1 2 3 4 5 6 7 1 2 3 4 5 6 7
N1 N3 T1 T2 T3
CpG site: 1 2 3 4 5 6 7 1 2 3 4 5 6 7 1 2 3 4 5 6 7 1 2 3 4 5 6 7 1 2 3 4 5 6 7
G
 T4 T5 T6 T7 T8
CpG site: 1 2 3 4 5 6 7 1 2 3 4 5 6 7 1 2 3 4 5 6 7 1 2 3 4 5 6 7 1 2 3 4 5 6 7

## Slide 2
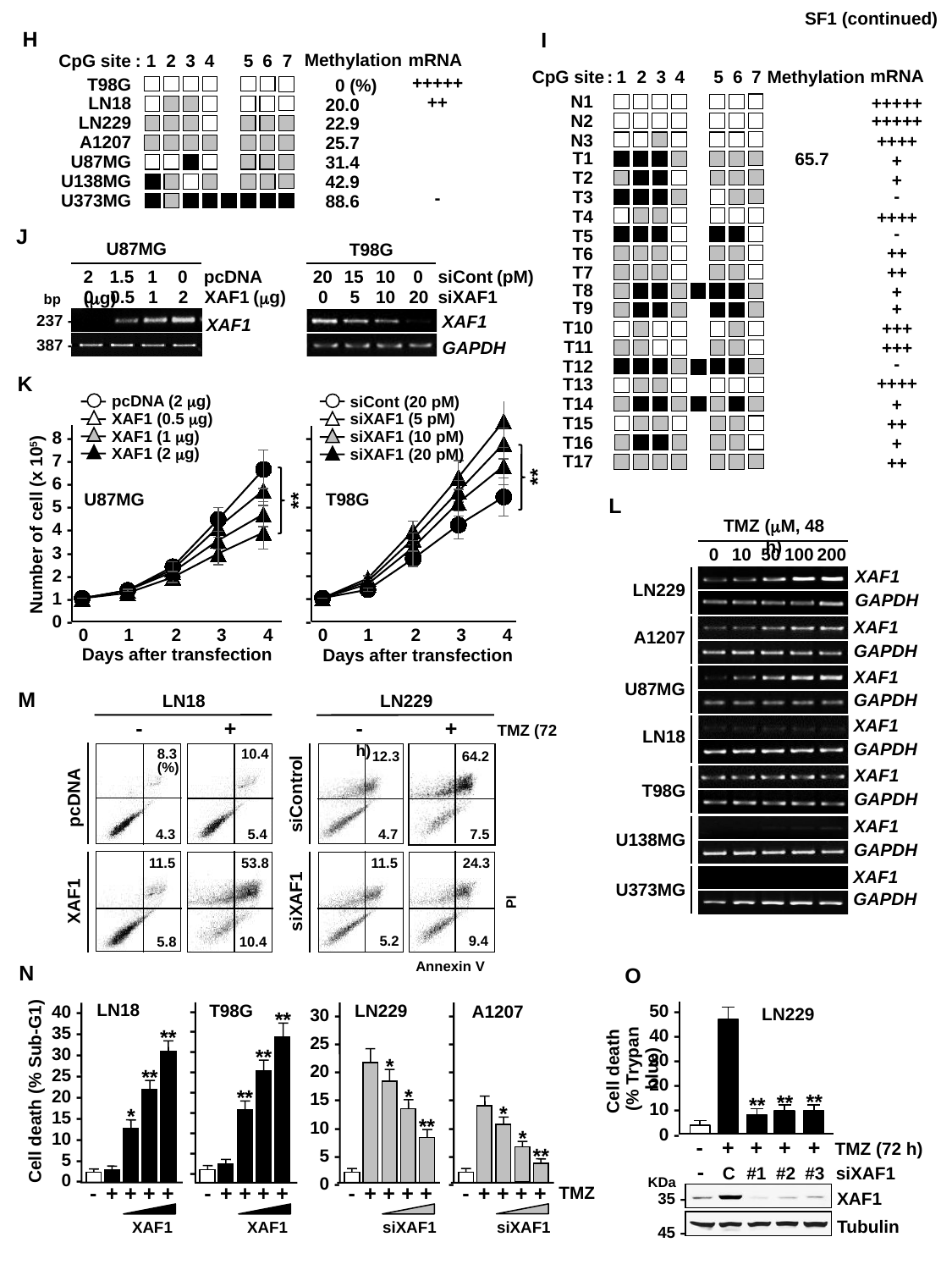

SF1 (continued)
H
I
CpG site : 1 2 3 4 5 6 7
mRNA
+++++
++
+++
++
++
+
-
Methylation
 0 (%)
 20.0
 22.9
 25.7
 31.4
 42.9
 88.6
T98G
LN18
LN229
A1207
U87MG
U138MG
U373MG
CpG site : 1 2 3 4 5 6 7
Methylation
 0 (%)
 0
 5.7
 65.7
 54.3
 68.6
 8.6
 60.0
 22.9
 25.7
 73.0
 62.9
 20.0
 17.1
 78.4
 11.4
 64.9
 28.6
 65.7
 27.6
mRNA
+++++
+++++
++++
+
+
-
++++
-
++
++
+
+
+++
+++
-
++++
+
++
+
++
N1
N2
N3
T1
T2
T3
T4
T5
T6
T7
T8
T9
T10
T11
T12
T13
T14
T15
T16
T17
J
U87MG
2 1.5 1 0 pcDNA (μg)
0 0.5 1 2 XAF1 (μg)
bp
XAF1
GAPDH
237 -
387 -
T98G
20 15 10 0 siCont (pM)
0 5 10 20 siXAF1 (pM)
XAF1
GAPDH
K
pcDNA (2 μg)
XAF1 (0.5 μg)
XAF1 (1 μg)
XAF1 (2 μg)
siCont (20 pM)
siXAF1 (5 pM)
siXAF1 (10 pM)
siXAF1 (20 pM)
### Chart
| Category | 계열 2 | 계열 3 | 계열 4 | 계열 5 |
|---|---|---|---|---|
| 0 | 0.0 | 0.0 | 0.0 | 0.0 |
| 0.4 | 0.7 | 0.6 | 0.5 | 0.3 |
| 2 | 2.4 | 2.1 | 1.8 | 1.4 |
| 3 | 4.3 | 3.8 | 3.4 | 2.6 |
| 4 | 6.3 | 5.5 | 4.7 | 3.6 |
 -
 -
 -
 -
 -
 -
 -
 -
 -
8 -
7 -
6 -
5 -
4 -
3 -
2 -
1 -
0 -
### Chart
| Category | 계열 2 | 계열 3 | 계열 4 | 계열 5 |
|---|---|---|---|---|
| 0 | 0.0 | 0.0 | 0.0 | 0.0 |
| 0.4 | 0.2 | 0.3 | 0.3 | 0.3 |
| 2 | 0.8 | 1.0 | 1.1 | 1.2 |
| 3 | 1.7 | 2.2 | 2.7 | 3.0 |
| 4 | 2.5 | 3.2 | 4.1 | 4.9 |
**
U87MG T98G
**
Number of cell (x 105)
0 1 2 3 4
0 1 2 3 4
Days after transfection
Days after transfection
L
TMZ (μM, 48 h)
0 10 50 100 200
XAF1
GAPDH
LN229
A1207
U87MG
LN18
XAF1
GAPDH
XAF1
GAPDH
XAF1
GAPDH
XAF1
GAPDH
T98G
U138MG
U373MG
XAF1
GAPDH
XAF1
GAPDH
M
LN229
LN18
- + TMZ (72 h)
- +
12.3 64.2
8.3 10.4
(%)
siXAF1 siControl
4.3 5.4
4.7 7.5
XAF1 pcDNA
11.5 53.8
11.5 24.3
PI
5.2 9.4
5.8 10.4
Annexin V
N
O
LN18
T98G
-
-
-
-
-
-
-
-
-
40 -
35 -
30 -
25 -
20 -
15 -
10 -
5 -
0 -
**
**
**
**
Cell death (% Sub-G1)
**
*
- + + + +
- + + + +
XAF1
XAF1
30 -
25 -
20 -
15 -
10 -
5 -
0 -
 -
 -
 -
 -
 -
 -
-
LN229
A1207
*
*
*
**
*
**
TMZ
- + + + +
- + + + +
siXAF1
siXAF1
50 -
40 -
30 -
20 -
10 -
0 -
LN229
Cell death
(% Trypan blue)
**
**
**
- + + + + TMZ (72 h)
- C #1 #2 #3 siXAF1
KDa
XAF1
Tubulin
35 -
45 -

## Slide 3
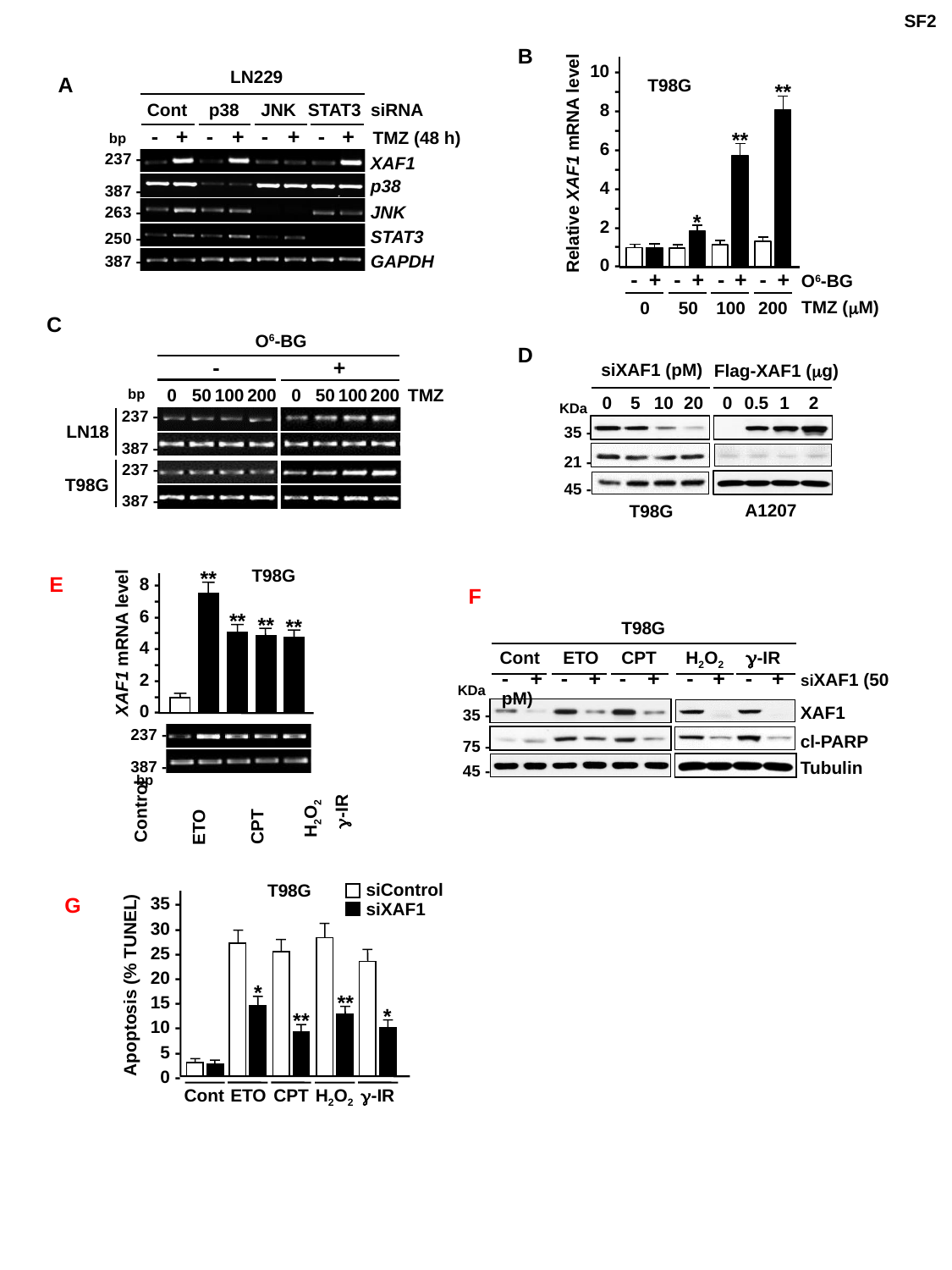

SF2
B
10 -
-
8 -
 -
6 -
 -
4 -
-
2 -
-
0 -
T98G
**
**
Relative XAF1 mRNA level
*
- + - + - + - + O6-BG
0 50 100 200
TMZ (μM)
LN229
Cont p38 JNK STAT3 siRNA
- + - + - + - + TMZ (48 h)
bp
XAF1
p38
JNK
STAT3
GAPDH
237 -
387 -
263 -
250 -
387 -
A
C
 O6-BG
- +
bp
0 50 100 200 0 50 100 200 TMZ (μM)
XAF1
GAPDH
237 -
387 -
LN18
T98G
XAF1
GAPDH
237 -
387 -
D
siXAF1 (pM)
Flag-XAF1 (μg)
0 5 10 20 0 0.5 1 2
KDa
XAF1
MGMT
Tubulin
35 -
21 -
45 -
A1207
T98G
**
T98G
8 -
-
6 -
-
4 -
-
2 -
-
0 -
**
**
**
XAF1 mRNA level
XAF1
GAPDH
237 -
387 -
Control
ETO
CPT
H2O2
γ-IR
bp
E
F
T98G
Cont ETO CPT H2O2 γ-IR
- + - + - + - + - + siXAF1 (50 pM)
KDa
XAF1
cl-PARP
Tubulin
35 -
75 -
45 -
T98G
siControl
siXAF1
35 -
30 -
25 -
20 -
15 -
10 -
5 -
0 -
Apoptosis (% TUNEL)
*
**
*
**
Cont ETO CPT H2O2 γ-IR
G

## Slide 4
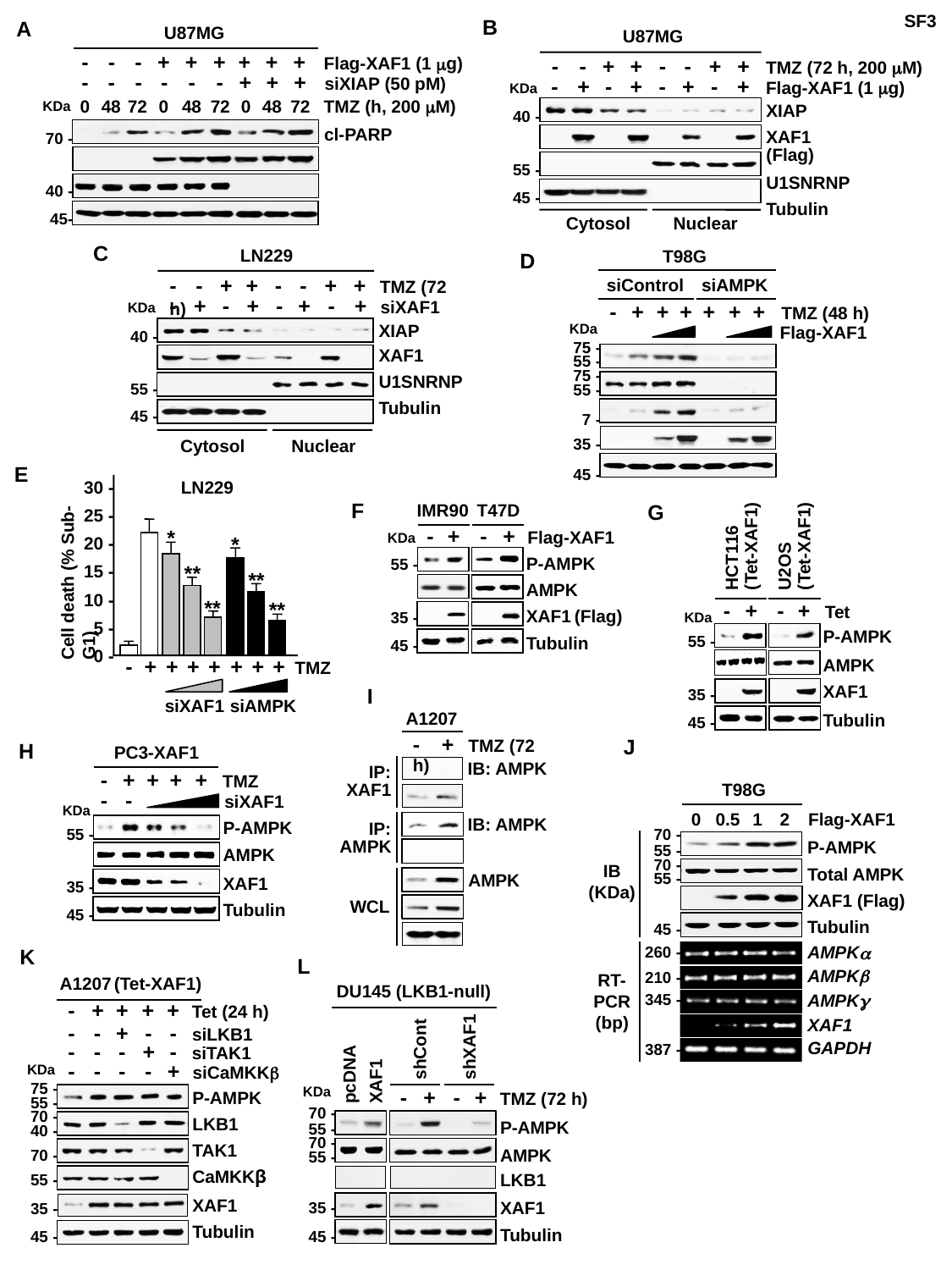

SF3
B
A
U87MG
- - - + + + + + + Flag-XAF1 (1 μg)
- - - - - - + + + siXIAP (50 pM)
0 48 72 0 48 72 0 48 72 TMZ (h, 200 μM)
cl-PARP
XAF1 (Flag)
XIAP
Tubulin
KDa
70 -
35 -
40 -
45-
U87MG
- - + + - - + + TMZ (72 h, 200 μM)
- + - + - + - + Flag-XAF1 (1 μg)
KDa
XIAP
XAF1 (Flag)
U1SNRNP
Tubulin
40 -
35 -
55 -
45 -
Cytosol Nuclear
C
LN229
- - + + - - + + TMZ (72 h)
- + - + - + - + siXAF1
KDa
XIAP
XAF1
U1SNRNP
Tubulin
40 -
35 -
55 -
45 -
Cytosol Nuclear
T98G
siControl siAMPK
 - + + + + + + TMZ (48 h)
 Flag-XAF1
KDa
P-AMPK
AMPK
cl-CASP3
XAF1 (Flag)
Tubulin
75 -
55 -
75 -55 -
7 -
35 -
45 -
D
E
30 -
25 -
20 -
15 -
10 -
5 -
0 -
LN229
*
*
Cell death (% Sub-G1)
**
**
**
**
- + + + + + + + TMZ
siXAF1
siAMPK
HCT116
(Tet-XAF1)
U2OS
(Tet-XAF1)
- + - + Tet
KDa
P-AMPK
AMPK
XAF1
Tubulin
55 -
55 -
35 -
45 -
F
G
IMR90 T47D
- + - + Flag-XAF1
KDa
P-AMPK
AMPK
XAF1 (Flag)
Tubulin
55 -
55 -
35 -
45 -
I
A1207
- + TMZ (72 h)
IB: AMPK
IB: XAF1
IP:
XAF1
IP:
AMPK
IB: AMPK
IB: XAF1
AMPK
XAF1
Tubulin
WCL
J
H
PC3-XAF1
- + + + + TMZ
- - siXAF1
KDa
P-AMPK
AMPK
XAF1
Tubulin
55 -
55 -
35 -
45 -
T98G
0 0.5 1 2 Flag-XAF1 (μg)
P-AMPK
Total AMPK
XAF1 (Flag)
Tubulin
70 -
55 -
70 -55 -
35 -
45 -
IB
(KDa)
RT-PCR
(bp)
260 -
210 -
345 -
237 -
387 -
AMPKα
AMPKβ
AMPKγ
XAF1
GAPDH
K
L
A1207 (Tet-XAF1)
- + + + + Tet (24 h)
- - + - - siLKB1
- - - + - siTAK1
- - - - + siCaMKKβ
P-AMPK
LKB1
TAK1
CaMKKβ
XAF1
Tubulin
KDa
75 -
55 -
70 -40 -
70 -
55 -
35 -
45 -
DU145 (LKB1-null)
shCont
shXAF1
pcDNA
XAF1
KDa
- + - + TMZ (72 h)
70 -
55 -
70 -55 -
40 -
35 -
45 -
P-AMPK
AMPK
LKB1
XAF1
Tubulin

## Slide 5
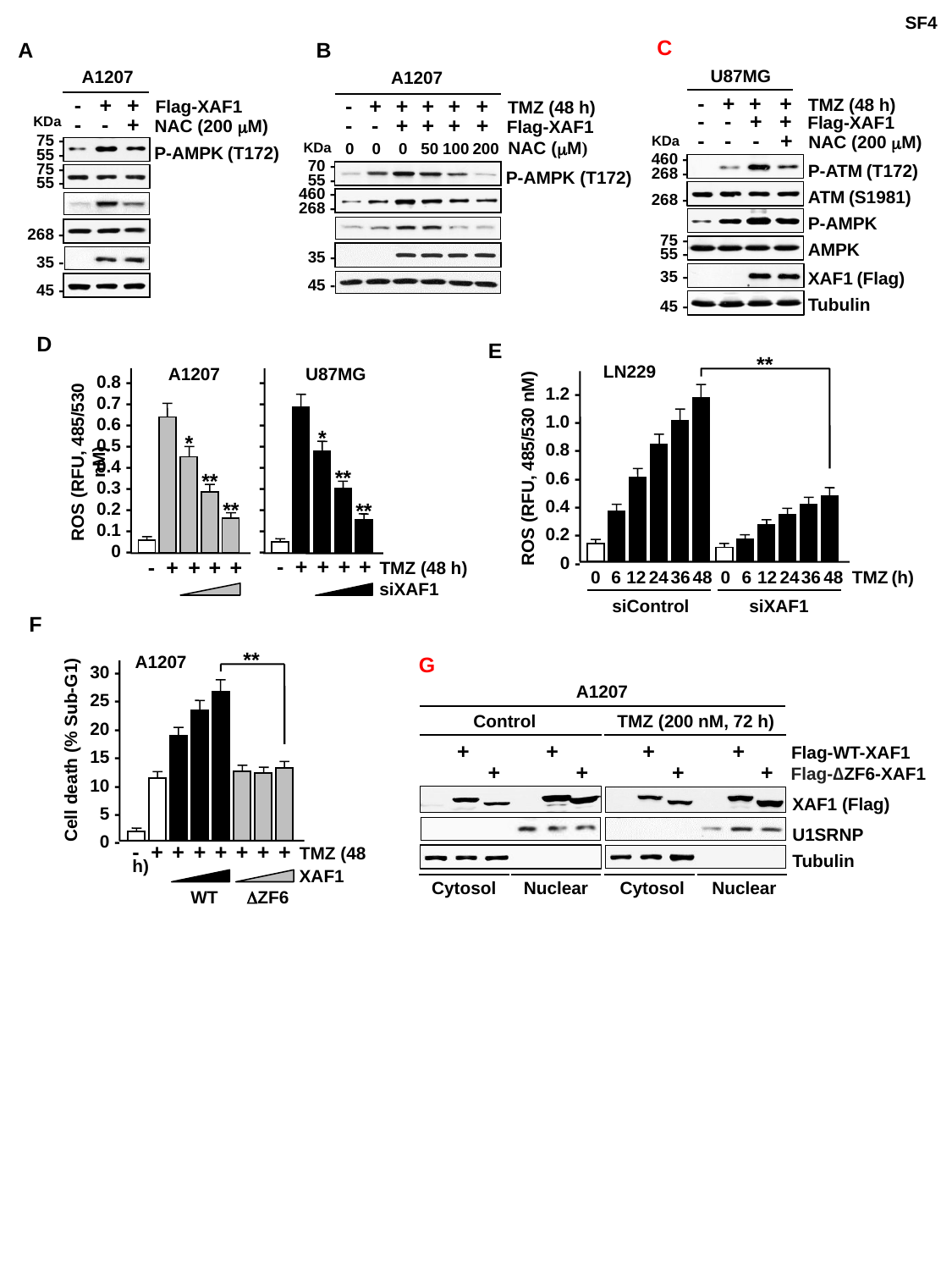

SF4
C
A
B
U87MG
- + + + TMZ (48 h)
- - + + Flag-XAF1
- - - + NAC (200 μM)
KDa
460 -
268 -
268 -
75 -
55 -
75 -
55 -
35 -
45 -
P-ATM (T172)
ATM (S1981)
P-AMPK
AMPK
XAF1 (Flag)
Tubulin
A1207
- + + Flag-XAF1
- - + NAC (200 μM)
KDa
75 -
55 -
75 -55 -
460 -
268 -
268 -
35 -
45 -
P-AMPK (T172)
AMPK
P-ATM (S1981)
ATM
XAF1 (Flag)
Tubulin
A1207
- + + + + + TMZ (48 h)
- - + + + + Flag-XAF1
0 0 0 50 100 200 NAC (μM)
KDa
70 -
55 -
460 -268 -
70 -
35 -
45 -
P-AMPK (T172)
P-ATM (S1981)
cl-PARP
XAF1 (Flag)
Tubulin
D
E
**
LN229
1.2 -
1.0 -
0.8 -
0.6 -
0.4 -
0.2 -
0 -
ROS (RFU, 485/530 nM)
0 6 12 24 36 48 0 6 12 24 36 48 TMZ (h)
siControl siXAF1
A1207
U87MG
 0.8 -
0.7 -
0.6 -
0.5 -
0.4 -
0.3 -
0.2 -
0.1 -
0 -
 -
-
-
-
-
-
-
-
-
*
*
ROS (RFU, 485/530 nM)
**
**
**
**
- + + + + TMZ (48 h)
- + + + +
siXAF1
F
A1207
**
30 -
25 -
20 -
15 -
10 -
5 -
0 -
Cell death (% Sub-G1)
- + + + + + + + TMZ (48 h)
XAF1
WT ΔZF6
G
A1207
Control TMZ (200 nM, 72 h)
 + + + + Flag-WT-XAF1
 + + + + Flag-∆ZF6-XAF1
XAF1 (Flag)
U1SRNP
Tubulin
Cytosol Nuclear
Cytosol Nuclear

## Slide 6
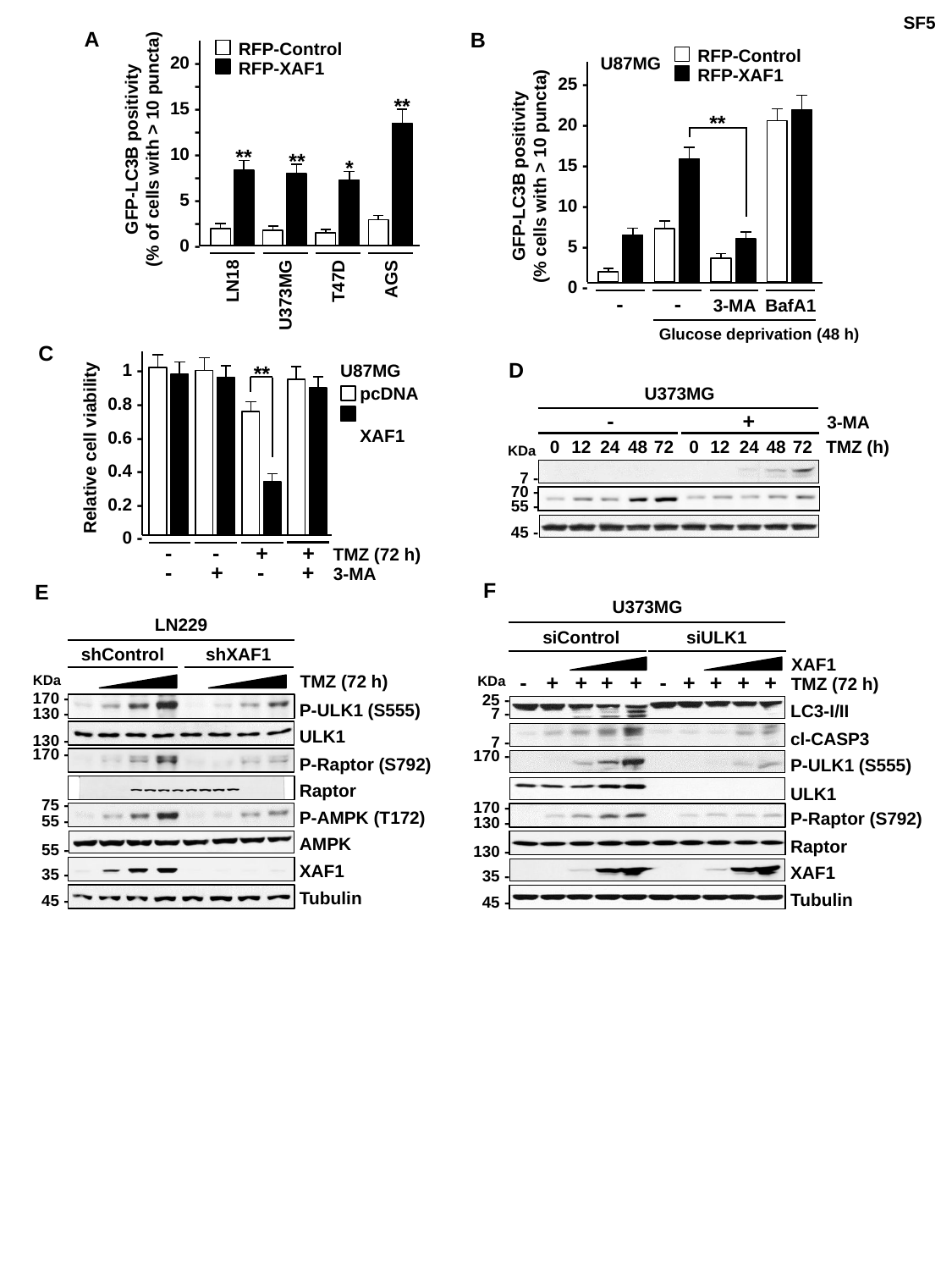

SF5
RFP-Control
RFP-XAF1
20 -
 -
15 -
 -
10 -
 -
5 -
-
0 -
**
GFP-LC3B positivity
(% of cells with > 10 puncta)
**
**
*
LN18
U373MG
T47D
AGS
A
B
RFP-Control
RFP-XAF1
25 -
20 -
15 -
10 -
5 -
0 -
U87MG
**
GFP-LC3B positivity
(% cells with > 10 puncta)
- - 3-MA BafA1
Glucose deprivation (48 h)
C
1 -
0.8 -
0.6 -
0.4 -
0.2 -
0 -
**
U87MG
pcDNA
XAF1
Relative cell viability
- - + + TMZ (72 h)
- + - + 3-MA
D
U373MG
- + 3-MA
0 12 24 48 72 0 12 24 48 72 TMZ (h)
KDa
cl-CASP3
Beclin-1
Tubulin
7 -
70 -
55 -
45 -
F
E
U373MG
siULK1
siControl
XAF1
KDa
- + + + + - + + + + TMZ (72 h)
25 -
7 -
7 -
170 -
130 -
130 -
170 -
130 -
130 -
35 -
45 -
LC3-I/II
cl-CASP3
P-ULK1 (S555)
ULK1
P-Raptor (S792)
Raptor
XAF1
Tubulin
LN229
shXAF1
shControl
TMZ (72 h)
KDa
170 -
130 -
130 -
170 -
130 -
130 -
75 -
55 -
55 -
35 -
45 -
P-ULK1 (S555)
ULK1
P-Raptor (S792)
Raptor
P-AMPK (T172)
AMPK
XAF1
Tubulin

## Slide 7
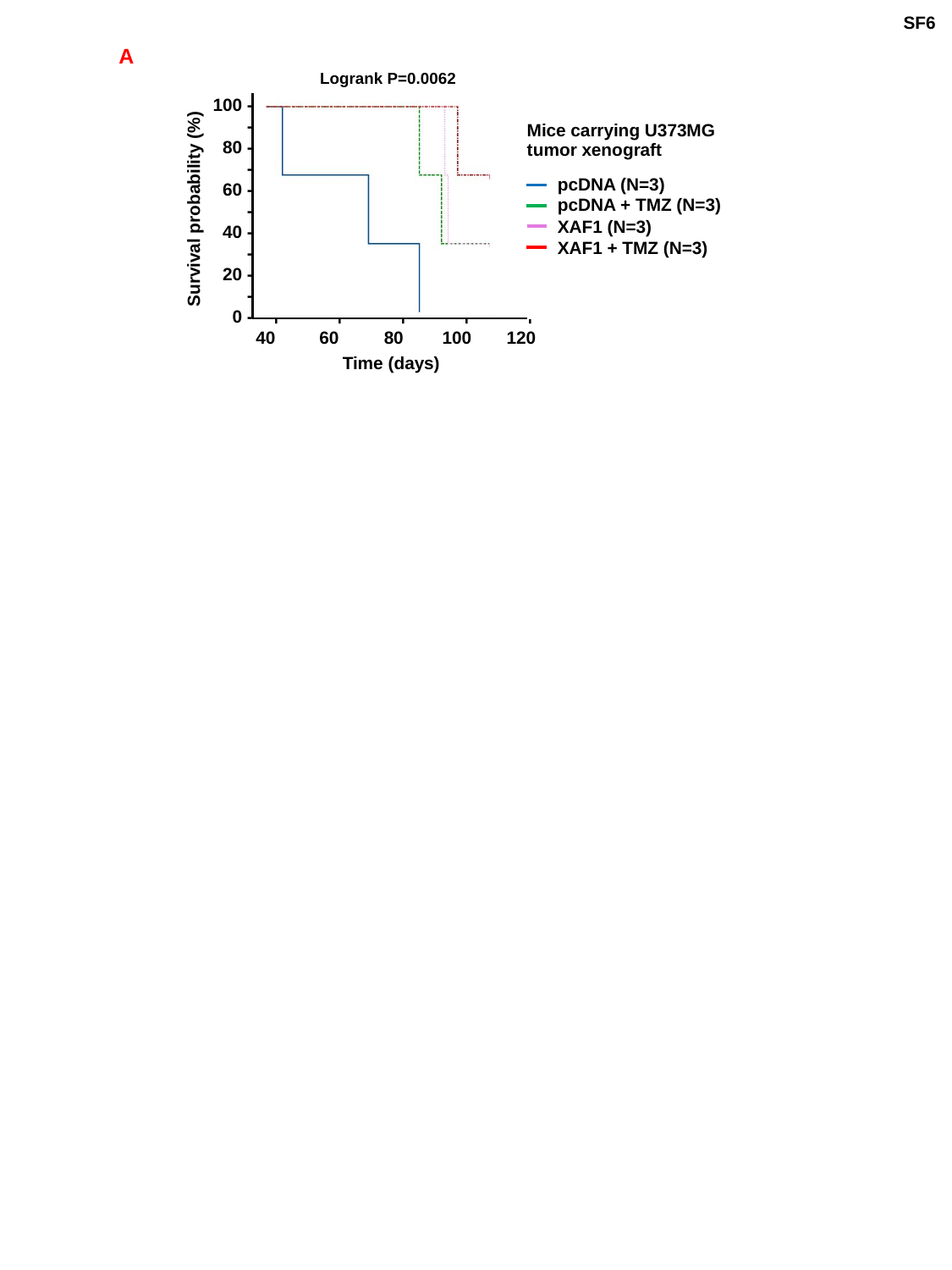

SF6
A
Logrank P=0.0062
100 -
-
80 -
-
60 -
-
40 -
-
20 -
-
0 -
Mice carrying U373MG
tumor xenograft
-
-
-
-
-
pcDNA (N=3)
pcDNA + TMZ (N=3)
XAF1 (N=3)
XAF1 + TMZ (N=3)
Survival probability (%)
40 60 80 100 120
Time (days)
