# Supplementary material for: XAF1 directs glioma response to temozolomide through apoptotic transition of autophagy by activation of ATM–AMPK signaling
Source: Neurooncol Adv. 2022 Feb 7;4(1):vdac013. doi: 10.1093/noajnl/vdac013 (PMC8903238; doi:10.1093/noajnl/vdac013)
Supplement: vdac013_suppl_Supplementary_Materials [file vdac013_suppl_supplementary_materials.docx]

**Supplementary Materials and Methods**

**Tumor tissues, Cancer Cell Lines and Reagents**

Tissue specimens were snap‑frozen immediately in liquid N_2_ and stored at ‑80°C until used. Bits of tumors and adjacent portions of each tumor were fixed and used for hematoxylin and eosin staining for histopathological evaluation. Signed informed consent was obtained from each patient. The A1207 (Tet‑XAF1) cells were generated by co‑transfection of XAF1 (pcDNA4/TO) and tetracycline repressor vector (pcDNA6/TR) (Invitrogen). T98G, U87MG, and LN229 sublines with shRNA-mediated knockdown of XAF1 were established by transfection of a shXAF1 construct (Genolution Pharmaceuticals Inc) and Zeocin (Invitrogen) selection. XAF1 stable sublines of U373MG were selected by G418 (200-600 μg/mL). Cyclohexamide (CHX), Compound C (CC), 3‑MA, O^6^‑BG, KU60019, and *N*‑acetylcysteine (NAC) were purchased from Sigma Aldrich. TMZ was purchased from Schering‑Plough. BafA1 was purchased from Millipore Sigma. For patient database analysis, the Cancer Genome Atlas (TCGA) database was analyzed using the MedCalc software (MedCalc Software Inc). The public RNA-seq data from GBM patients were analyzed and the overall survival results were plotted with the mRNA expression of *XAF1* and *MGMT*. The data for the Kaplan-Meier curve was obtained from web-based tool KM Plotter (http://www.kmplot.com). Log-rank *p*-value and Hazard ratio (HR) were calculated with a Mantel-Cox test and represented with 95% confidential intervals.

**Semi‑Quantitative Polymerase Chain Reaction (PCR), 5‑Aza‑dC Treatment and Bisulfite DNA Sequencing Analysis**

Total cellular RNA (1 μg) was converted to cDNA by reverse transcription using random hexamer primers and reverse transcriptase (M‑MLV, Thermo Fisher Scientific). PCR was performed over a range of cycles (28-36 cycles) by using 1:4 diluted cDNA (12.5 ng per 50 μl of PCR) with primers used for *XAF1* (sense; 5ʹ‑CAGAAGTCCTCGCTGGAGTTTC‑3ʹ and antisense; 5ʹ‑TGAAATTCTTTCCCCTTTCC ‑3ʹ), *AMPKα* (sense; 5ʹ‑CATATAATTAAACTGTAC‑3ʹ and antisense; 5ʹ‑AGAATAACCCCACTGCTC‑3ʹ), *AMKPβ* (sense; 5ʹ‑CTGGAGCGGCATGCTGGC‑3ʹ and antisense; 5ʹ‑GGGAGATAAGTAAACTTC‑3ʹ), *AMPKγ* (sense; 5ʹ‑CATCGCTGCTATGACCTG‑3ʹ and antisense; 5ʹ‑CAAGCTGGCATTAGGAGA‑3ʹ), and an endogenous expression standard gene *GAPDH*. PCR products were resolved on 2% agarose gels and quantitated by densitometric scanning of the ethidium bromide‑stained gels. Integration and analysis were performed by using Quantity One software program (Bio-Rad). For methylation assay, cells were treated with 5 μM of 5‑Aza‑dC (Sigma-Aldrich) for 4 days and *XAF1* mRNA expression was evaluated by RT‑PCR. For bisulfite DNA sequencing analysis, 50 ng of bisulfite‑modified DNA was subjected to PCR amplification of the promoter region comprised of CpG sites. The PCR products were cloned into pCRII vectors (Invitrogen) and 5 clones of each specimen were sequenced by automated fluorescence‑based DNA sequencing to determine the methylation status.

**Immunoblotting, Immunoprecipitation and Immunohistochemistry**

Antibodies specific for XAF1 (SC‑19194), AMPK (CST #5831), P‑AMPK (CST #2531), LKB1 (CST #3050), TAK1 (SC‑7967), CaMKKβ (SC‑271674), ATM (CST #2873), P‑ATM (CST #5883), XIAP (CST #2045), LC3‑I/II (CST #4108), ULK (CST #8054), P‑ULK (CST #14202), Raptor (CST #2280), P‑Raptor (CST #2083), cleaved PARP (CST #9541), CASP3 (CST #9665), cleaved CASP3 (CST #9661S), U1SNRNP (SC‑390899), and β‑tubulin (T0198) were purchased from Santa Cruz Biotechnology, Cell Signaling Technology, Abcam, or BD Bioscience. Immunohistochemistry was carried out using the Bond Polymer Intense Detection System (VisionBio Systems). Briefly, 4 μm sections of paraffin‑embedded tissues were incubated for 15 min with monoclonal XAF1 antibody (Santa Cruz Biotechnology) using biotin‑free polymeric horseradish peroxidase‑linker antibody conjugate system. The slides were washed with OBS and incubated with avidin‑biotin‑peroxidase complex for 1 h. The color was developed with kit substrate solution. For the immunoreactive score, we established as 1-to-5-point system by multiplying the percentage of positive cells by the intensity of the staining score.

**Transfection, Cell Growth, Luciferase Reporter and Chromatin Immunoprecipitation (ChIP) Assays**

Small interfering RNA (siRNA) duplex against *XAF1* ('5ʹ‑AUGUUGUCCAGACUCAGAG‑3ʹ), *AMPK‑α1* (5′‑GCAGAAGUAUGUAGAGCAA‑3′), *MGMT* (4255‑1), and *IRF-1* (5ʹ-CAGAUUAAUUCCAACCAA-3ʹ) were synthesized by Bioneer Inc. siRNAs against *ATM* (CST #6328), *TAK1* (CST #6317), *ULK1* (CST #7000), and *CAMKKβ* (110916) were synthesized by Cell Signaling Technology or Invitrogen. Control siRNA duplex served as a negative control was purchased from Dharmacon Research. Transfection of siRNAs or expression plasmids was performed using Neon® Transfection System (Invitrogen), Lipofectamine 2000 (Invitrogen) or Turbofect™ in vitro Transfection Reagent (Thermo Fisher Scientific). For cell number counting assay, cells (2 x 10^4^) were seeded in six‑well plate in triplicate. The cells were transfected with expression vector or siRNA and cell numbers were counted using a hemocytometer at 24‑h intervals. For flow cytometry analysis, cells were fixed with 70% ethanol and resuspended in 1 ml of PBS containing 50 mg/ml RNase and 50 mg/ml propidium iodide (Sigma). The assay was performed on a FACScan flow cytometer (Becton Dickinson), and the cell cycle profile was analyzed using MultiCycle software (Phoenix Flow Systems). For colony formation assay, cells (1 x 10^5^) were maintained for 10 days. Colonies were fixed with methanol for 15 min and stained with 0.5% crystal violet in 20% ethanol. The *XAF1* promoter region was cloned into the pGL3‑basic vector (Promega). Putative IRF‑1 binding site was mutated using site‑directed mutagenesis. Cells were transfected with 500 ng of reporter constructs using Lipofectamine 2000 (Invitrogen). After normalization of each extract for protein content, luciferase activity was measured by using the Luciferase assay system (Promega). ChIP assay was carried out using a Simple ChIP™ Enzymatic Chromatin IP Kit (Cell signaling) and antibodies specific for IRF‑1. PCR was done using primers F‑10 (sense, 5ʹ‑AAAAGGGATGGAGACCCAGA‑3ʹ) and P2 (antisense, 5ʹ‑CACACCGAGAAGTCTCCTTC‑3ʹ) for the *XAF1* promoter region comprising IRF‑1 binding site (IRFE).

**ROS Measurement and Mitochondria Membrane Permeability**

For microscopic analysis, cells were incubated with 10 µM of 2'-7'dichlorofluorescin diacetate (DCFH‑DA, Molecular Probes) for 30 min, and fluorescence images were taken using an inverted microscope (U‑RFLT50/CK40, Olympus). For microplate analysis, cells were seeded in poly L‑lysine pre‑coated 96 well black bottom culture plates. After incubation with DCFH‑DA (20 µM, 30 min), ROS level was monitored by measuring the fluorescence intensity using a microplate reader (SpectraMax i3x, Molecular Devices) with excitation and emission wavelengths set at 485 nm and 528 nm, respectively. The mitochondrial membrane potential (ΔΨm) was determined by using flow cytometric analysis of JC-1 (tetraethyl-benzimidazolylcarbo-cyanine iodide, Molecular Probes). Briefly, cells (1 X 10^5^) were incubated with 250 nM of JC-1 dye for 30 min. The cells were washed and resuspended with PBS. Using FACS Calibur (BD Biosciences) and CellQuest Research software, the red fluorescence emitted by JC-1 accumulated in the mitochondria membrane was measured at emission of 525 nm (Fl1; JC1 green) and 585 nm (Fl2, JC1 red) from excitation at the 488 nm.

**Immunofluorescence and Microscopic Autophagy Assays**

U87MG cells were seeded in 2‑well chamber slide (154461, Thermo Fisher Scientific) and co‑transfected with GFP‑LC3B and either red fluorescent protein (RFP)-control or RFP-XAF1. The cells were treated with TMZ (100 μM) for 48 h. Compound C (10 μM), 3-MA (5 μM), or BafA1 (100 nM) were added 2 h before TMZ treatment. The cells were fixed in 4% paraformaldehyde for 10 min and fluorescent images of cells showing punctuated changes of GFP‑LC3 were obtained with the confocal microscope (LSM510META, Carl Zeiss AG). A minimum of 150 transfected cells were counted to quantify the puncta signals for each sample.

For autophagy detection, cells were seeded on glass chamber slides (Lab-Tek 177429, Nalgene Nunc) and transfected with expression vectors encoding a green fluorescent protein (GFP), GFP-WT-XAF1 and GFP-ΔZF6-XAF1. After 48 h incubation, the cells were fixed with 4% (vol/vol) paraformaldehyde-Tris HCl-buffered saline (TBS), permeabilized with 0.5% Triton X-100, and blocked with 5% (wt/vol) BSA for 1 h. MitoTracker^TM^ (Thermo Fisher Scientific) was used for the counterstaining of the mitochondria. After the final wash with TBS, coverslips were mounted with Prolong Gold antifade reagent (Molecular Probes). Fluorescent imaging was examined using a confocal laser-scanning microscope (LSM700, Carl Zeiss MicroImaging, Inc) and LSM 510 software.

**Animal Studies**

Four‑week‑old immunodeficient male nude mice (nu/nu) (Orient Bio Inc.) were maintained in pressurized ventilated cages. Briefly, the identical numbers (4 x 10^6^) of pcDNA-U373MG and XAF1-U373MG subline cells were injected into two groups of mice (n = 6 per group). Tumor growth was monitored periodically, and volume (V) was calculated by using the modified ellipsoidal formula: V = 1/2 × length × (width)^2^. At days 20 and 25, three mice of each group were exposed to saline or temozolomide (40 mg/kg) by oral gavage, and tumor volume was measured at the beginning of injection and monitored regularly for 65 days. A survival analysis was carried out using the identical set of mice. All studies were performed with the approval of Korea University Institutional Animal Care and Use Committee and Korea Animal Protection Law.
